# The Stable Association of Virion with the Triple-gene-block Protein 3-based Complex of *Bamboo mosaic virus*

**DOI:** 10.1371/journal.ppat.1003405

**Published:** 2013-06-06

**Authors:** Yuan-Lin Chou, Yi-Jing Hung, Yang-Hao Tseng, Hsiu-Ting Hsu, Jun-Yi Yang, Chiung-Hua Wung, Na-Sheng Lin, Menghsiao Meng, Yau-Heiu Hsu, Ban-Yang Chang

**Affiliations:** 1 Institute of Biochemistry, National Chung-Hsing University, Taichung, Taiwan, Republic of China; 2 Biotechnology Center, National Chung-Hsing University, Taichung, Taiwan, Republic of China; 3 Institute of Plant and Microbial Biology, Academia Sinica, Taipei, Taiwan, Republic of China; 4 Graduate Institute of Biotechnology, National Chung-Hsing University, Taichung, Taiwan, Republic of China; IBMP CNRS Université de Strasbourg, France

## Abstract

The triple-gene-block protein 3 (TGBp3) of *Bamboo mosaic virus* (BaMV) is an integral endoplasmic reticulum (ER) membrane protein which is assumed to form a membrane complex to deliver the virus intracellularly. However, the virus entity that is delivered to plasmodesmata (PD) and its association with TGBp3-based complexes are not known. Results from chemical extraction and partial proteolysis of TGBp3 in membrane vesicles revealed that TGBp3 has a right-side-out membrane topology; i.e., TGBp3 has its C-terminal tail exposed to the outer surface of ER. Analyses of the TGBp3-specific immunoprecipitate of Sarkosyl-extracted TGBp3-based complex revealed that TGBp1, TGBp2, TGBp3, capsid protein (CP), replicase and viral RNA are potential constituents of virus movement complex. Substantial co-fractionation of TGBp2, TGBp3 and CP, but not TGBp1, in the early eluted gel filtration fractions in which virions were detected after TGBp3-specific immunoprecipitation suggested that the TGBp2- and TGBp3-based complex is able to stably associate with the virion. This notion was confirmed by immunogold-labeling transmission electron microscopy (TEM) of the purified virions. In addition, mutational and confocal microscopy analyses revealed that TGBp3 plays a key role in virus cell-to-cell movement by enhancing the TGBp2- and TGBp3-dependent PD localization of TGBp1. Taken together, our results suggested that the cell-to-cell movement of potexvirus requires stable association of the virion cargo with the TGBp2- and TGBp3-based membrane complex and recruitment of TGBp1 to the PD by this complex.

## Author Summary

Plant viruses spread their infectious entities from cell to cell via plasmodesmata (PD) through the assistance of virus-encoded movement proteins and host factors. Some RNA viruses encode three functionally coordinated movement proteins organized into a triple gene block (TGB) to facilitate their cell-to-cell movement. TGBp2 and TGBp3 are known to associate with the endoplasmic reticulum (ER) membrane and ER-derived vesicles. The ER- or vesicle-associated TGBp2 and TGBp3 presumably form a membrane complex to deliver the viruses. However, the identity of the “viral RNA cargo” and whether the cargo is able to associate with the TGBp2- and TGBp3-containing membrane complex during intracellular transport remain unclear for potex-like viruses. Taking advantage of an HA-tagged and a His-tagged TGBp3 construct of *Bamboo mosaic virus* (BaMV), we have been able to determine the membrane topology of TGBp3, isolate the TGBp3-based complex and detect the existence of a stable TGBp2-TGBp3-virion complex. Moreover, we have clarified that TGBp3 plays a key role in virus cell-to-cell movement by enhancing the TGBp2- and TGBp3-dependent PD localization of TGBp1. These results suggested that the cell-to-cell movement of potexvirus requires stable association of the virion cargo with the TGBp2- and TGBp3-containing membrane complex and recruitment of TGBp1 to the PD by this complex.

## Introduction

Plant viruses spread their genomes from infected cell to uninfected cell via plasmodesmata (PD) with the assistance of virus-encoded movement proteins. Some RNA viruses, like those in *Virgaviridae* [1] and *Flexiviridae* [2] families, encode three movement proteins organized into a triple gene block (TGB) to facilitate cell-to-cell and long distance movement of viruses. According to phylogenetic comparisons and differences in the mechanism of movement, the TGB-encoding viruses or the TGB proteins are classified into hordei-like and potex-like classes [3].

*Bamboo mosaic virus* (BaMV) belonging to the potex-like class has a monopartite, positive sense, single-stranded RNA genome with a 5′ cap and a 3′ poly(A) tail. The five open reading frames (ORFs) between the 5′ and 3′ untranslated regions of the BaMV genome encode the 155-kDa replicase, 28-kDa TGBp1, 13-kDa TGBp2, 6-kDa TGBp3 and 25-kDa CP, respectively [4]. The three TGB proteins are absolutely required for virus movement [5]. Functional mechanisms of the TGB proteins have been investigated. TGBp1 is located at the cytoplasm and nuclei in the form of inclusions in BaMV- and PVX-infected tissues [6], [7]. The cytoplasmic TGBp1 inclusions release functional TGBp1 with ATP-binding, ATPase, and RNA-binding activities, and the efficiency of TGBp1 release is significantly enhanced by the presence of vRNA and CP [8]–[11]. Moreover, TGBp1 has helicase activity [12] and the ability to increase the size exclusion limit (SEL) of PD [13]–[15] as well as to promote translation of virus-derived RNAs [16]–[18]. TGBp2 is an integral membrane protein of endoplasmic reticulum (ER) and ER-derived granular vesicles [7], [19]–[23] with both its N- and C-terminal tails exposed to the cytosol [19] and it is able to bind vRNA in a non-specific manner *in vitro* [24]. Ala substitutions of the two conserved Cys residues at the C-terminal tail of TGBp2 make the cell-to-cell movement of BaMV relatively inefficient and systemic movement severely inhibited [25]. TGBp3 was found to reside in the ER at the same location of replicase [26]. Moreover, TGBp3 is able to interact with TGBp2 as ectopically co-expressed in yeast and to target TGBp2 to the cortical ER tubules of cell periphery by the sorting signal in the C-terminal region of TGBp3 in both yeast and plant [27], [28]. The interactions among the TGB proteins and CP in various combinations, like TGBp2-TGBp1, TGBp2-CP, TGBp3-TGBp2-TGBp1 and TGBp3-TGBp2-CP, have been observed using bimolecular fluorescence complementation (BiFC) assay in yeast [28]. However, the relation of these interactions to virus movement remains unclear.

Detailed models have been proposed to describe how hordei-like and potex-like viruses move from cell to cell [29], [30]. In hordei-like viruses, the TGBp1 encapsidated viral (v)RNA first associates with the TGBp2- and TGBp3-based complex on the endoplasmic reticulum (ER) network to form a virus movement complex which is then directed to PD by the PD-targeting signal of TGBp3. CPs are dispensable in the process. In potex-like viruses, both TGBp2-based and TGBp3-driven processes have been proposed. In the TGBp2-based process, two pathways have been considered. In the first pathway, TGBp1 interacts with virion or virion-like particle to form a complex which is transported to and across the PD. In the second pathway, TGBp2 induces novel vesicles containing TGBp3 to bud from the ER. The vesicles associate with actin and move toward the PD; however, it is uncertain whether they are able to mediate the transport of vRNA cargo. In the TGBp3-driven process, TGBp2 and TGBp3 colocalized in the ER are proposed to drive viral genome, either in the form of virion (virion or TGBp1-virion) or non-virion ribonucleoprotein (RNP) complex (TGBp1-vRNA-CP) to peripheral membrane bodies (PMB) through the trafficking signals of TGBp3 and across the PD through the assistance of a TGBp2-interacting protein (TIP) and β-1,3-glucanase. CPs are indispensable in both the TGBp2-based and TGBp3-driven processes. Nevertheless, it is unclear whether the TGBp2- and TGBp3-based complex in the ER or ER-derived vesicles is able to directly mediate transport of vRNA cargo.

This study was aimed to identify the “vRNA cargo” and to clarify whether it is able to associate with the TGBp3-containing membrane complex. In other words, we aimed to identify the main components of virus movement complex during intracellular transport of potexvirus. To fulfill this goal, we determined the membrane topology of BaMV TGBp3 having a C-terminal HA or His-tag fusion and isolated the TGBp3-based complex from membrane fraction of infected tissues through Sarkosyl extraction. Results from gel filtration analysis of the Sarkosyl extract, analyses of protein components in the TGBp3-specific immunoprecipitate of gel filtration samples and immunogold-labeling transmission electron microscopy of purified BaMV virions demonstrated that the virion can be the “vRNA cargo” that stably associates with the TGBp2- and TGBp3-containing membrane complex. Moreover, mutational and confocal microscopy analyses revealed that TGBp3 plays a key role in virus cell-to-cell movement by enhancing the TGBp2- and TGBp3-dependent PD localization of TGBp1.

## Results

### The C-terminal HA fusion of TGBp3 does not have any negative effect on both cell-to-cell and long-distance movement of BaMV

**Figure 1:**
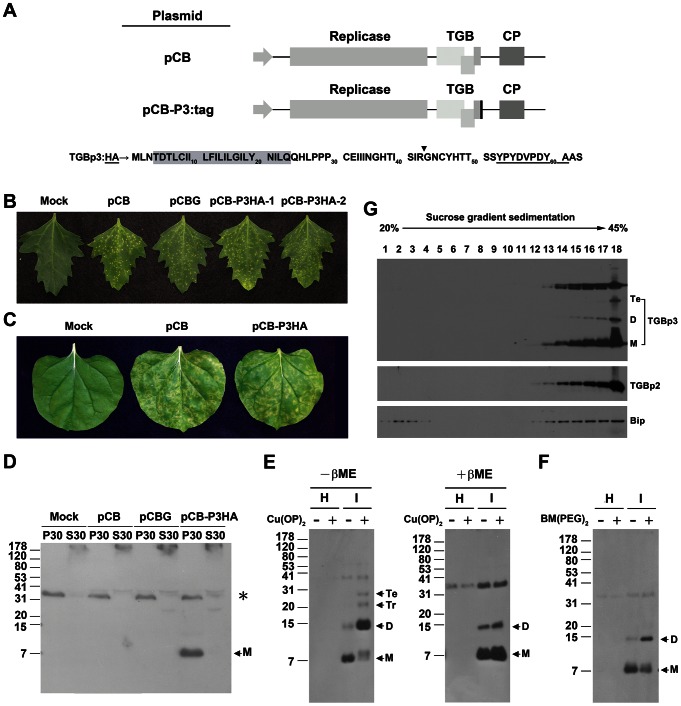
Construction of BaMV expressing TGBp3 with a C-terminal HA fusion and characterization of the TGBp3:HA. (**A**) Linear maps of BaMV genome in pCB and pCB-P3:tag. The five open reading frames (ORFs) of BaMV-S in pCB and pCB-P3:tag are driven by the 35S promoter (gray arrow). The three partially overlapping ORFs, designated as TGB, encode TGBp1, TGBp2 and TGBp3, respectively, from left to the right. The additional black box at the 3′ end of the TGBp3 coding region in pCB-P3:tag indicates the tag fused at the C-terminal end of TGBp3. Shown below the linear maps is the amino acid (aa) sequence of TGBp3:HA. The amino acid residues in dark gray box constitute the transmembrane region of TGBp3; the downward triangle indicates the unique trypsin cleavage site in TGBp3; the amino acid sequence of HA tag is underlined. The two amino acid residues, Ala (A) and Ser (S) following the HA tag are encoded by the sequence of *Nhe* I site. (**B**) Disease symptom of pCB-, pCBG- or pCB-P3HA-infected *C. quinoa* leaf. pCB-P3HA-1 and pCB-P3HA-2 are two BaMV clones containing an HA tag at the C-terminal end of TGBp3. (**C**) Disease symptom of the systemic leaf of *N. benthamiana* as infected by pCB or pCB-P3HA. (**D**) Fractionation of TGBp3:HA in the membrane fraction, P30, of the pCB-P3HA-infected tissues as probed with monoclonal anti-HA antibody. M, the monomeric form of TGBp3:HA. (**E**) Effect of Cu(OP)_2_ on multimerization of TGBp3:HA. βME is the abbreviation of β-mercaptoethanol. − and + indicate electrophoresis of the P30 protein samples in the absence or presence of βME, respectively. The numbers on the left margin are the relative migration positions of prestained protein markers of different molecular masses (in kDa) on Tricine SDS-polyacrylamide gel. (**F**) The effect of BM(PEG)_2_ on multimerization of TGBp3:HA. In both (**E**) and (**F**), H and I indicate P30 prepared from healthy and infected *Nicotiana benthamiana* leaves, respectively. M, D, Tr, and Te indicate monomeric, dimeric, trimeric and tetrameric forms of TGBp3:HA, respectively. (**G**) Co-fractionation of TGBp3:HA with ER marker proteins, TGBp2 and BiP. The numbers labeled on top of the panel indicate the fraction number from top to bottom of the 20 to 45% linear sucrose gradient.

The study was aimed to isolate and characterize the movement complex of BaMV. To fulfill this goal, a co-immunoprecipitation strategy was adopted. TGBp3 which assists the targeting of the ER-localized TGBp2 or the TGBp2-containing vesicles to cell periphery [20], [21], [27] and thus the movement of virus across cells [28], [29] was chosen as the primary target for co-immunoprecipitation. However, the preparation of anti-TGBp3 using the hydrophilic region of TGBp3 or the His-tagged TGBp3 as immunogen was unsuccessful. Thus, other small tag such as HA or Flag was fused to the C-terminus of TGBp3 through recombinant DNA technique (Figure 1A), hoping that the anti-tag could enable us to immunologically detect TGBp3 and co-immunoprecipitate the virus movement complex after infection of the host with the recombinant plasmid clone of BaMV, pCB-P3:tag. Luckily, pCB-P3HA expressing TGBp3:HA had an infectivity similar to that of its wild-type (Wt) counterpart, pCB, on *Chenopodium quinoa* (Figure 1B), indicating that TGBp3:HA is able to assist cell-to-cell movement of BaMV. However, the C-terminal fusion of TGBp3 with a Flag tag made BaMV lose its infectivity similar to that observed for the fusion of TGBp3 with GFP or mCherry (data not shown). Moreover, TGBp3:HA did not affect long distance movement of BaMV as evidenced by the presence of a severe disease symptom in the systemic leaves of *Nicotiana benthamiana* (Figure 1C).

### TGBp3 is an integral membrane protein with its C-terminal tail exposed to the outer surface of membrane structure

To immunologically detect TGBp3:HA, the *N. benthamiana* leaves infected with pCB-P3HA were first separated into P1 (cell wall, organelle and cell debris), S30 (cytoplasm) and P30 (membrane) fractions and then probed with monoclonal anti-HA. TGBp3:HA in monomeric form was clearly detected in P30 of the pCB-P3HA-infected *N. benthamiana* but not in S30 and the control P30 prepared from that inoculated with mock, pCB or pCBG (pCB with a green fluorescence protein gene) (Figure 1D). Thus, TGBp3:HA is mainly associated with the membrane fraction. However, an anti-HA cross-reacting signal (as indicated by *) migrated a bit slower than the 31-kDa marker protein was also detected.

It has been reported that BaMV TGBp2 has the ability to oligomerize [19]. To see whether TGBp3 also has the ability to oligomerize, effect of redox condition on multimerization of TGBp3:HA in P30 prepared from BaMV-infected tissues was first performed. As shown in Figure 1E, significant increase in dimeric, trimeric and tetrameric TGBp3:HA were observed when the P30 was treated with the oxidation reagent, Cu(OP)_2_, and solubilized with sample application buffer containing 9 M urea but lacking reducing agent, β-mercaptoethanol, before being electrophoresed on Tricine SDS-polyacrylamide gel (left panel). However, the relative level of dimeric TGBp3:HA decreased significantly as β-mercaptoethanol was incorporated into the sample application buffer (right panel). Additionally, multimerization of TGBp3:HA in the presence of a membrane-impermeable bismaleimide crosslinker, BM(PEG)_2_, was tested. As shown in Figure 1F, an increase in the level of dimeric TGBp3:HA was observed. Taken together, these results indicated that TGBp3:HA proteins in the membrane fraction (P30) prepared from the BaMV-infected tissues are able to associate with each other to form oligomers.

**Figure 2:**
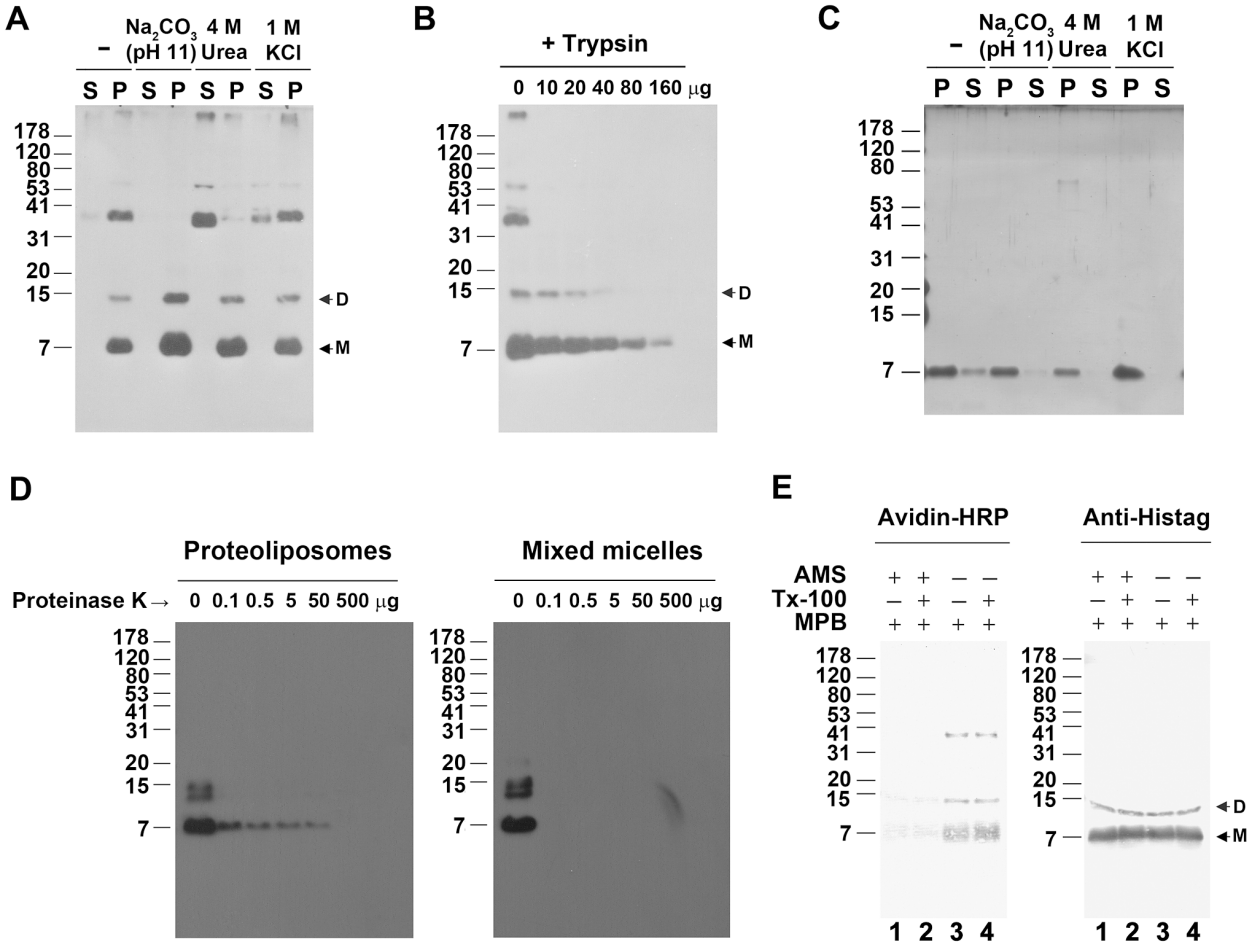
Membrane topology of BaMV TGBp3. (**A**) Chemical extraction of TGBp3:HA in membrane. The TGBp3:HA-containing membrane sample (P30) were extracted with Na_2_CO_3_ (pH 11), 4 M urea or 1 M KCl, and centrifuged at 30,000 ***g*** to separate the supernatant (S30) and pellet (P30). S and P indicate S30 and P30, respectively. (**B**) Partial proteolysis of TGBp3:HA in membrane vesicles with trypsin. The protein products from partial proteolysis was subjected to Tricine SDS-PAGE, transferred to PVDF membrane and probed with monoclonal anti-HA. D and M, the dimeric and monomeric TGBp3:HA, respectively. (**C**) Chemical extraction of TGBp3:H6 in *in vitro* reconstituted proteoliposomes. P, TGBp3:H6 in pellet after centrifugation. S, TGBp3:H6 in supernatant fraction. (**D**) Partial proteolysis of TGBp3:H6 in *in vitro* reconstituted proteoliposomes. Left and right panels indicate digestion of TGBp3:H6 in reconstituted proteoliposomes untreated and pre-treated with 1% Triton X-100, respectively. (**E**) Chemical modification of TGBp3:HA in *in vitro* reconstituted proteoliposomes. The maleimides used for sequential modification of cysteine residues (Cys-32 and Cys-47) in the C-terminal tail of TGBp3:H6 are 4-acetamido-4′-maleimidylstilbene-2, 2′-disulfonic acid disodium salt (AMS) and *N*_α_-(3-maleimidylpropionyl) biocytin (MPB). Left panel, the MPB-modified signal of TGBp3:H6 as detected by avidin-HRP. Right panel, the signal of TGBp3:H6 as detected by anti-His tag.

To identify the membrane domain with which TGBp3:HA associates, co-fractionation of TGBp3:HA with two ER marker proteins, BiP and TGBp2, was examined by linear sucrose density gradient (20 to 45%) centrifugation, with which both marker proteins are mainly associated with rough ER and sediment at higher sucrose concentration [19], [31]. As shown in Figure 1G, TGBp3:HA closely co-fractionated with both TGBp2 and BiP at higher sucrose concentration, indicating that TGBp3 is mainly associated with ER. To clarify that TGBp3 is an integral or a peripheral membrane protein, the TGBp3:HA-containing membraneous extracts (P30) and the *in vitro* reconstituted proteoliposomes containing exclusively TGBp3:H6 as described in Figures S1 and S2 were treated with Na_2_CO_3_ (pH 11), 4 M urea or 1 M KCl. The treatment of membraneous extracts or *in vitro* reconstituted proteoliposomes with Na_2_CO_3_ (pH 11) was expected to release proteins entrapped within the membrane [32], and treatments with 4 M urea or 1 M KCl would dislodge proteins peripherally bound to lipid bilayers [33]. As shown in Figures 2A and 2C, TGBp3:HA and TGBp3:H6 remained associated with P30 and with the *in vitro* reconstituted proteoliposomes (P) after each of the extraction, indicating that TGBp3 is integrated into the lipid bilayers of membrane structure.

To analyze the membrane topology of TGBp3, trypsin digestion of TGBp3:HA in membraneous extracts (P30) was first performed. Since both the HA-tag and the sole trypsin-cleavage site, Arg-43, are located in the C-terminal tail region of TGBp3:HA (Figure 1A), the detection of a gradual decrease in HA signal by increasing the dose of trypsin (Figure 2B) indicated that the C-terminal tail of TGBp3:HA is exposed to the outer surface of the membrane structure. Moreover, we favored that TGBp3:HA in the membraneous extract (P30) possesses a right-side-out membrane topology because the same topology was obtained for the marker protein, TGBp2, under the same condition of membrane sample preparation [19], [34]. In parallel, the TGBp3:H6 in *in vitro* reconstituted proteoliposomes were subjected to partial proteolysis and examination with anti-His-tag. Consistent with the results of Figure 2B, the His-tag signal of TGBp3:H6 gradually disappeared as an increasing amount of proteinase K was added into the digestion mixture (Figure 2D, left panel); while the His-tag signal of TGBp3:H6 was quickly eliminated as the proteoliposomes were treated with TritonX-100 (Figure 2D, right panel). These results indicated that the C-terminal tail of TGBp3:H6 is exposed to the outer surface of proteoliposome. To further confirm this idea, sequential maleimide modification assay [19], [35] was performed. AMS and MPB are the two maleimides employed. MPB contains a biotin moiety which can be easily detected by avidin-HRP. If the two cysteine residues (Cys-32 and Cys-47) in the C-terminal tail of TGBp3:H6 are exposed to the outer surface of membrane, they would be pre-modified with AMS, which in turn would lower the level of subsequent modification of TGBp3:H6 with MPB. As shown in the left panel of Figure 2E, the MPB-modified signal of TGBp3:H6 was insignificant as the proteoliposomes were pretreated with AMS, no matter whether Triton X-100 was present or not (lanes 1 and 2). However, the MPB-modified signal of TGBp3:H6 was significantly increased as AMS was omitted (lanes 3 and 4). According the above results and the similarity in the level of TGBp3:H6 in each of the assayed samples (Figure 2E, right panel), we concluded that TGBp3 have its C-terminal tail exposed to the outer surface of the membrane structure.

### Extraction of the TGBp3-based complex with Sarkosyl

**Figure 3:**
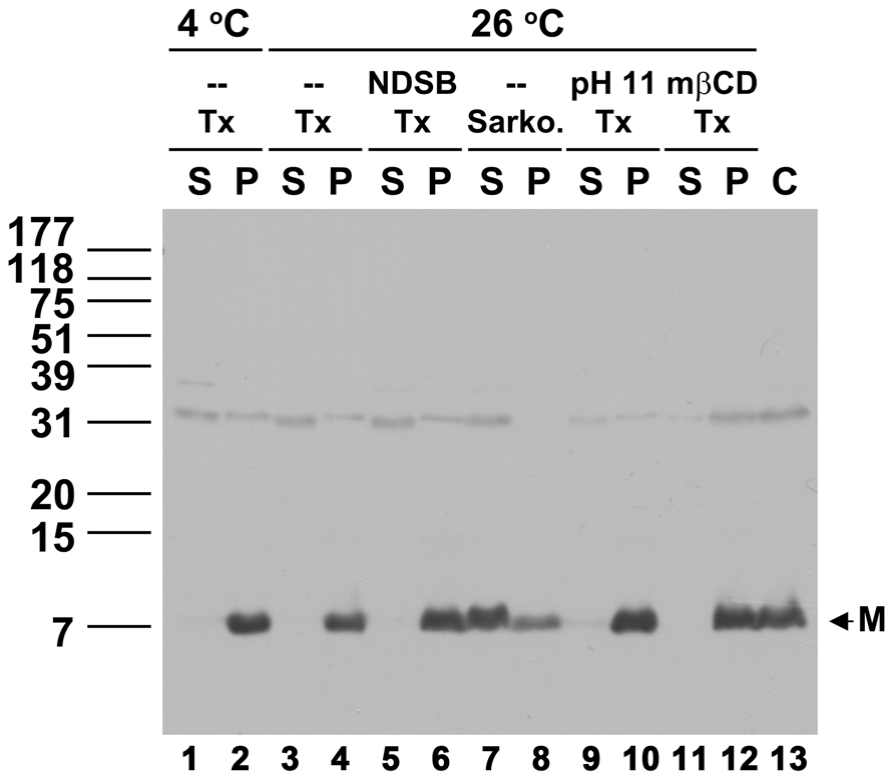
Effects of detergent and additive on extraction of the TGBp3:HA-containing membrane protein complex from P30. The TGBp3-containing membrane protein complexes in P30 were extracted with Triton X-100 (Tx) at 26°C in the presence or absence of high pH (pH 11), NDSB and mβCD, or extracted with 1% Sarkosyl (Sarko.). P and S indicate the supernatant and pellet samples, respectively, after extraction of the P30 with detergent and ultracentrifugation at 100,000 ***g***. C, the TGBp3:HA in P30 before extraction. M, the monomeric TGBp3:HA.

The membrane integration of TGBp3 led us to presume that extraction of the TGBp3-based complex from the ER or ER-derived membrane vesicles with a compatible detergent would enable us to isolate the virus movement complex if the TGBp3-based complex really serves as a vehicle for virus movement [29]. However, non-ionic detergent such as n-Dodecyl-β-maltoside (DDM), IGEPAL CA-630 or Triton X-100 failed to extract TGBp3-based complex from P30 (data not shown). Thus, non-detergent sulfobetaines (NDSBs) additive [36], methyl β-cyclodextrin (mβCD) [37], [38], pH or higher temperature, which may assist the extraction of membrane protein complex, was incorporated into the Triton X-100 extraction system. However, no TGBp3:HA was able to be extracted from P30 (P) to S100 (S) after treatment with higher temperature (Figure 3, lanes 3 and 4), NDSBs (lanes 5 and 6), pH (lanes 9 and 10) or mβCD (lanes 11 and 12), indicating that extraction of TGBp3-based protein complex from P30 remained inefficient after the above-mentioned treatments. The inefficiency of TGBp3 extraction, we thought, might be due to co-precipitation of the Triton X-100 extracted TGBp3-based protein complex with certain existing large complexes during ultracentrifugation, since the concentration of Triton X-100 (1%) used for the extraction was much higher than the critical micellar concentration of Triton X-100 (0.015%). To avoid the precipitation problem, an ionic detergent, Sarkosyl, used to purify BaMV RNA-capping enzyme and assay the endogenous RNA-dependent-RNA polymerase (RdRp) activity of BaMV [39], [40] was adopted. Fortunately, most of the TGBp3:HA signal or TGBp3:HA-based complex was extracted from P30 and retained in S100 after ultracentrifugation (Figure 3, lanes 7 and 8).

### Association of TGBp1, TGBp2, CP, replicase and vRNA with the TGBp3-based complex

**Figure 4:**
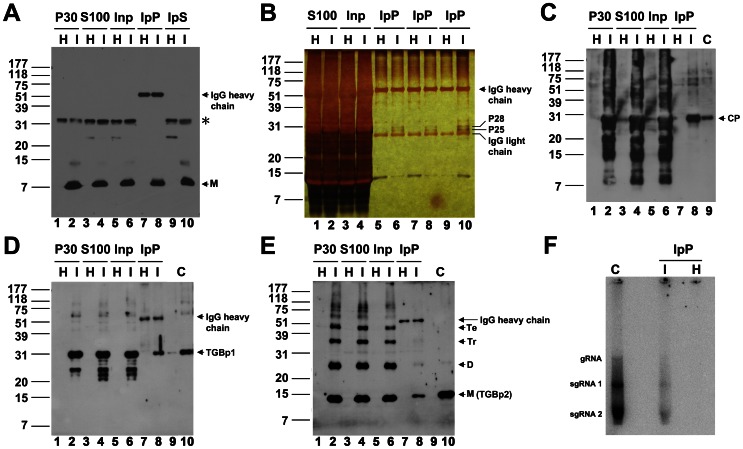
Co-immunoprecipitation of various virus components with TGBp3:HA. (**A**) Immunoprecipitation of the TGBp3:HA-containing membrane protein complex. Inp, the S100 prepared from mock-inoculated (H) and BaMV-infected tissues (I) pre-cleaned with Protein A-Sepharose (PA). IpP and IpS, the immunoprecipitate and supernatant, respectively, obtained through immunoprecipitation of Inp with anti-HA (see Materials and Methods). M, the monomeric TGBp3:HA. (**B**) Examination of protein components in the IpP. P25 and P28 are the two proteins detected only in the IpP prepared from the BaMV-infected tissues (I) but not from the mock-inoculated (H) tissues. (**C**–**E**) Co-immunoprecipitation of CP, TGBp1 and TGBp2 with TGBp3:HA, respectively. C indicates purified CP, TGBp1 or TGBp2 used as reference protein for western blot. M, D, Tr and Te shown on the right margin of panel E indicate the monomeric, dimeric, trimeric and tetrameric TGBp2. (**F**) Analysis of endogenous viral RNA and replicase in the IpP prepared from the BaMV-infected tissues. The positions of genomic (g)RNA, subgenomic (sg)RNA 1 and sgRNA 2 on the RNA gel are shown on the left margin. C, virus replication in the presence of 1.5% NP-40 solubilized P30 fraction which contains BaMV vRNA and replicase.

To identify the proteins associated with the Sarkosyl-extracted TGBp3:HA-based protein complex, immunoprecipitation of TGBp3:HA in the complex with anti-HA was first performed (Figure 4A). To begin with, the S100 samples (lanes 3 and 4) prepared from the healthy (H) and BaMV-infected (I) tissues were pre-cleaned with Protein A-Sepharose to generate Inp, the sample used for immunoprecipitation (lanes 5 and 6). After immunoprecipitation of the Inp with anti-HA, a significant portion of TGBp3:HA was found to reside in both IpP, the immunoprecipitate (lane 8) and IpS, the supernatant (lane 10). However, the cross-reacting signal (as indicated by *) migrating more slowly than the 31-kDa marker protein remained in IpS (lanes 9 and 10). These results indicated that the immunoprecipitation is specific for TGBp3:HA. Therefore, we further examined the proteins specifically co-immunoprecipitated with TGBp3:HA (IpP) by silver staining after Tricine SDS-PAGE (Figure 4B). Two proteins, P28 and P25 (lanes 6, 8 and 10), absent from the immunoprecipitate prepared in parallel from healthy leaves (lanes 5, 7, and 9), were detected. Both proteins were recovered from the gel, subjected to LC-MS/MS analysis (see Text S1) and identified to be TGBp1 and CP, which are essential for BaMV movement. To see whether there were other viral components associated with the TGBp3-based complex, the IpP was further subjected to western blotting (Figures 4C–E) and RdRp assay (Figure 4F). In western blotting, CP (Figure 4C, lane 8), TGBp1 (Figure 4D, lane 8) and TGBp2 (Figure 4E, lane 8) were detected. The detection of CP and TGBp1 was consistent with data obtained from LC-MS/MS analysis. In RdRp assay (Figure 4F), similar patterns of RNA transcripts were observed for replication with IpP prepared from infected tissues and with positive control (C), the 1.5% NP-40 solubilized P30 fraction containing BaMV replicase and endogenous vRNA [41]. These results indicated that there are endogenous vRNA and replicase in the IpP. Taken together, our results suggested that TGBp2, TGBp1, CP, vRNA and replicase are associated with the TGBp3-based complex.

### Stable association of BaMV virion with the TGBp2- and TGBp3-based complex

**Figure 5:**
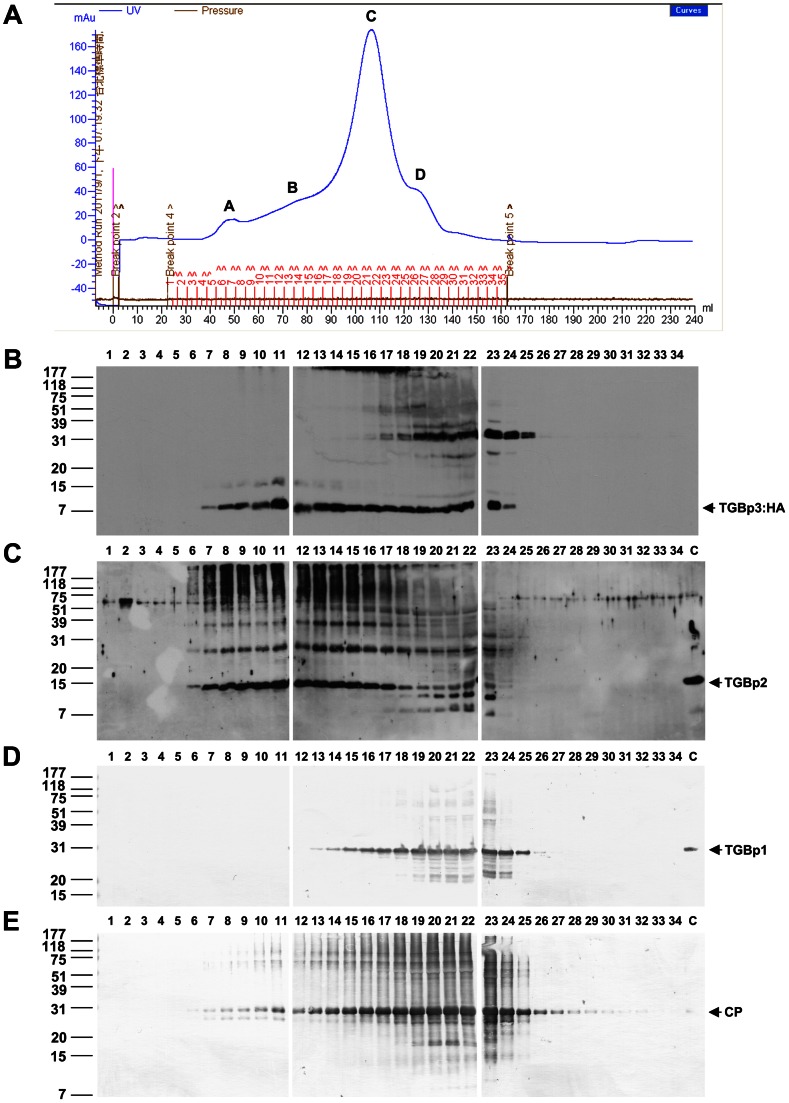
Co-fractionation of TGBp3, TGBp2 and CP along the Sephacryl S-1000 gel filtration profile. (**A**) The gel filtration profile of the S100 fractionated by the Sephacryl S-1000 column. A, B, C and D are the four major peaks detected in the profile; 1 to 34 are fraction numbers. (**B**–**E**) Immunological analyses of TGBp3:HA, TGBp2, TGBp1 and CP, respectively, in the samples fractionated by the Sephacryl S-1000 column. In **B** and **C**, protein bands were detected using a chemiilumination system. In D and E, protein bands were detected using 1-chloro-4-naphathol as a color development agent.

**Figure 6:**
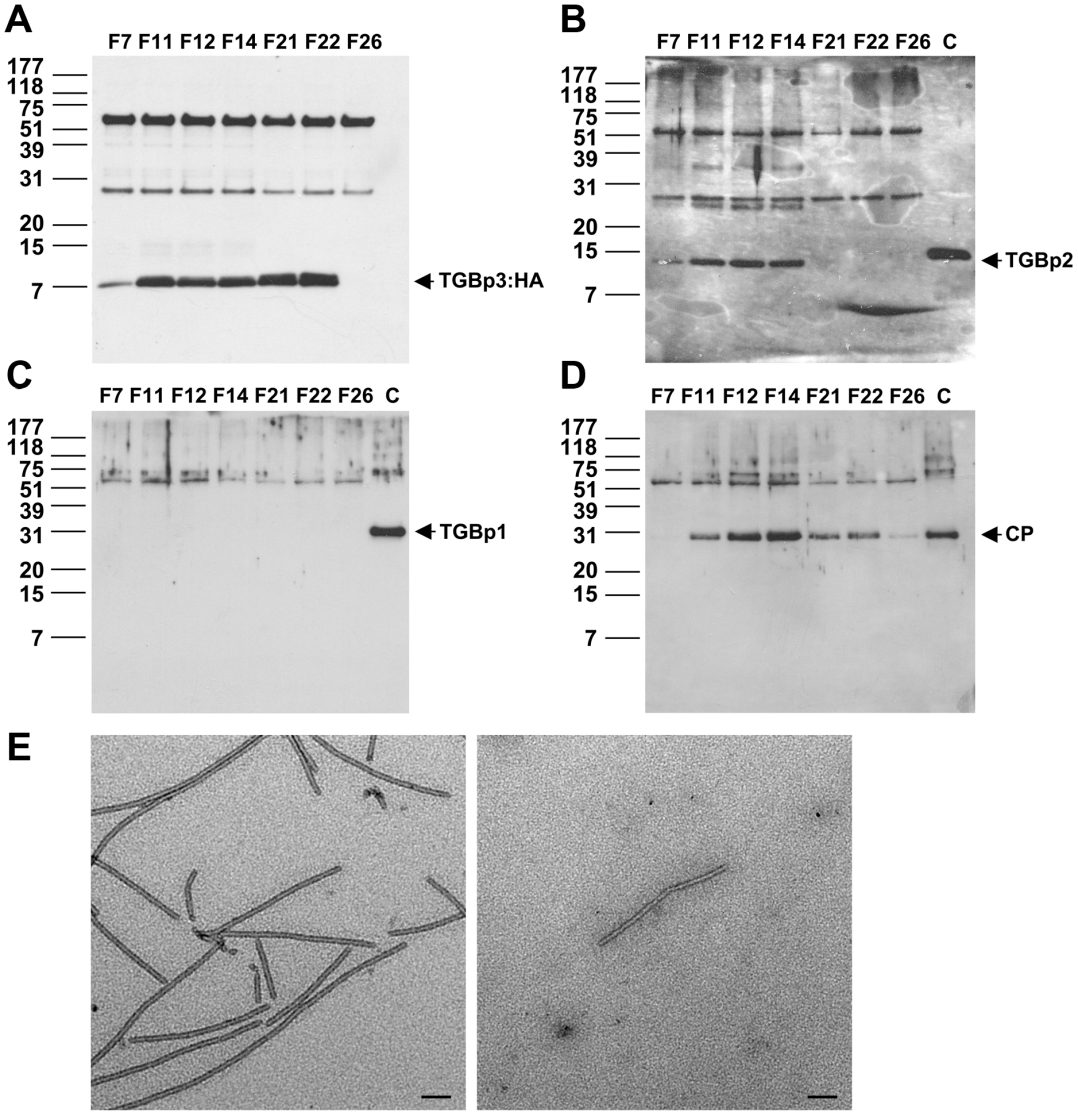
Association of the TGBp2- and TGBp3-based complex with CP on virions. The Sephacryl S-1000 fractionated S100 samples in peak A (F7), peak B (F11, 12, and 14), peak C (F21 and F22), and peak D (F26) as shown in Figure 5 were subjected to immunoprecipitation with anti-HA and the immunoprecipitates were analyzed immunologically with anti-HA (**A**), anti-TGBp2 (B), anti-TGBp1 (C), and anti-CP (D), respectively. (**E**) Co-immunoprecipitation of BaMV virion with TGBp3:HA as analyzed by transmission electron microscopy (TEM). Left panel, the purified BaMV-S virions. Right panel, the BaMV virion co-immunoprecipitated with TGBp3:HA in F12. Bars, 100 nm.

To further analyze the TGBp3-based complex, the Sarkosyl extracted fraction (S100) was applied onto a Sephacryl S-1000 gel filtration column suitable for separation of large complexes with molecular masses up to 10^8^ (100 MDa) or for separation of spherical particles up to 400 nm. The distributions of TGBp1, TGBp2, TGBp3 and CP along the filtration profile were analyzed by western blot (Figure 5). Apparently, substantial co-fractionation of TGBp3, TGBp1 and CP was observed in the largest peak, peak C. However, substantial co-fractionation of the main protein components required for intracellular virus transport, TGBp2 and TGBp3, and the early eluted CP (all peaked apparently at F11), but not TGBp1, was mainly observed in peak B. These results suggested to us that the TGBp2- and TGBp3-based complex is able to stably associate with the CP-coated virions but not non-virion vRNP which is assumed to possess TGBp1 [29]. To corroborate this idea, three sample fractions (F11, F12 and F14) from peak B and four (F7, F21, F22 and F26) from peaks A, C and D, respectively, were subjected to immunoprecipitation with anti-HA. The immunoprecipitate was then analyzed with anti-TGBp1, anti-TGBp2, anti-HA and anti-CP (Figure 6). As shown in Figure 6A, TGBp3:HA was able to be immunoprecipitated with anti-HA in all the tested fractions, except F26 (peak D). Significant co-immunoprecipitation of TGBp2 (Figure 6B) with TGBp3:HA was mainly significantly detected in fractions (F11, F12 and F14) of peak B. Significant co-immunoprecipitation of CP (Figure 6D) was observed in fractions (F11, F12, F14, F21 and F22) of peaks B and C. However, TGBp1 was unable to be co-immunoprecipitated with TGBp3:HA in all of the tested fractions (Figure 6C). Taken together, these results suggested that peak B contains TGBp2-TGBp3-based complex and BaMV virions rather than non-virion vRNP. In other words, the TGBp2-TGBp3-based complex may be able to associate with CP on the virions. To confirm this idea, the immunoprecipitate was examined with transmission electron microscopy (TEM). Just as expected, virus particles similar to the purified BaMV-S (Figure 6E, left panel) were observed in the immunoprecipitate (Figure 6E, right panel), indicating that the TGBp2-TGBp3-based complex is able to stably associate with virions.

### Association of BaMV virions with the TGBp2-TGBp3-based complex as analyzed by immunogold-labeling (IGL) TEM

**Figure 7:**
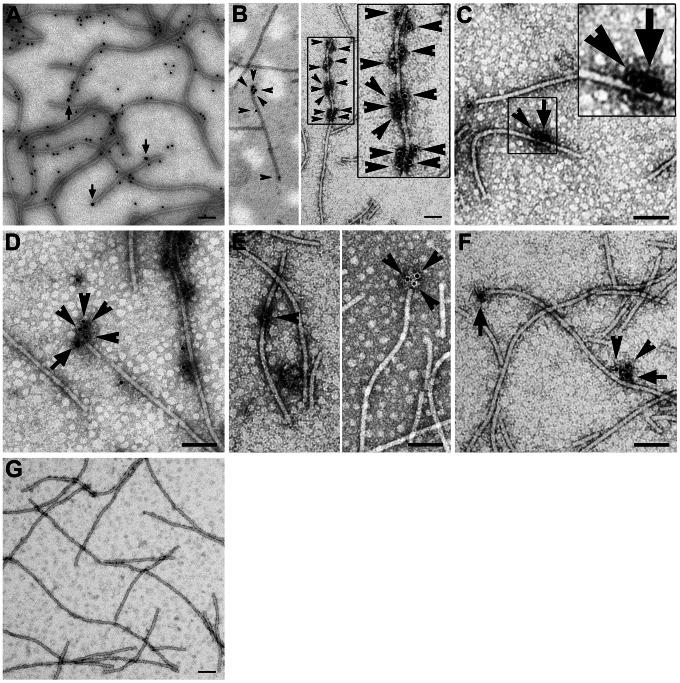
Association of TGBp3, TGBp2 and TGBp1 with BaMV virion. (**A**) Association of TGBp3:HA with BaMV virions purified from the pCB-P3HA-infected leaves. The arrows point to the positions of TGBp3:HA bound with anti-HA recognized by the 18 nm gold-conjugated secondary antibody and the 12 nm gold particles indicate CP on BaMV virions. (**B**) Association of TGBp2 with BaMV virions. The arrowheads point to the positions of TGBp2. (**C–D**) Association of the TGBp3- and TGBp2-containing protein complex with BaMV virions. TGBp3 (indicated by arrows) and TGBp2 (indicated by arrowheads) were located in cluster on BaMV virions. (**E**) Association of TGBp1 with BaMV virions. TGBp1 or TGBp1 cluster as indicated by arrowheads was located at the body or end of BaMV virions. (**F**) Association of the TGBp3- and TGBp1-containing protein complex with BaMV virions. TGBp3 as indicated by arrow and TGBp1 as indicated by arrowheads were located in cluster on BaMV virions. (**G**) Negative control for IGL procedure. The analysis was performed in the presence of 12 and 18 nm gold-conjugated secondary antibody but absence of any primary antibody. Bars, 100 nm.

To confirm the stable association of virions with the TGBp2-TGBp3-based complex, we purified the BaMV virions from the pCB-P3HA-infected leaves using a method with Triton X-100 extraction (see Material and Methods). We thought that the Triton X-100-extracted TGBp2-TGBp3-based complexes could be precipitated with the virions if they are truly able to stably associate with virions. The purified virions were thus analyzed with immunogold-labeling (IGL) TEM (Figure 7). In this analysis, the 18 nm colloidal gold-conjugated secondary antibody was specific for the recognition of anti-HA and thus the localization of TGBp3:HA; while the 12 nm colloidal gold-conjugated secondary antibody was specific for the recognition of either anti-CP, anti-TGBp2 or anti-TGBp1 and thus the localization of CP, TGBp2 or TGBp1, correspondingly. As a result, TGBp3:HA (the 18 nm particles as indicated by arrows) appeared to locate at the ends and both sides of the purified virions (Figure 7A), indicating that the virions are truly associated with the TGBp3-based complex. TGBp2 appeared to cluster at the ends and both sides of the virions (Figure 7B), indicating that they associate with the virions in multimeric form. Moreover, TGBp3 and TGBp2 were detected in the same virion at the same cluster (Figures 7C and 7D), indicating that the virions are able to associate with the TGBp2-TGBp3-based complexes. Based on the results of immunoprecipitation and immunogold-labeling TEM as shown above, we concluded that the TGBp2-TGBp3-based complex and the virions are able to form a stable complex in BaMV-infected tissues.

We further analyzed whether TGBp1 is able to associate with the stable complex of TGBp3-TGBp2-virion, since the association of TGBp1 with the TGBp3-based complex was also observed during immunoprecipitation of the S100 prior to gel filtration (Figure 4). As shown in Figure 7, TGBp1 appeared at the body and end of the virions (Figure 7E), or located at one side of the virions in association with TGBp3 (Figure 7F), indicating that TGBp1 is able to associate with the stable complex of TGBp3-TGBp2-virion under a certain condition.

### Both TGBp2 and TGBp3 are required for TGBp1 targeting to the PD

**Figure 8:**
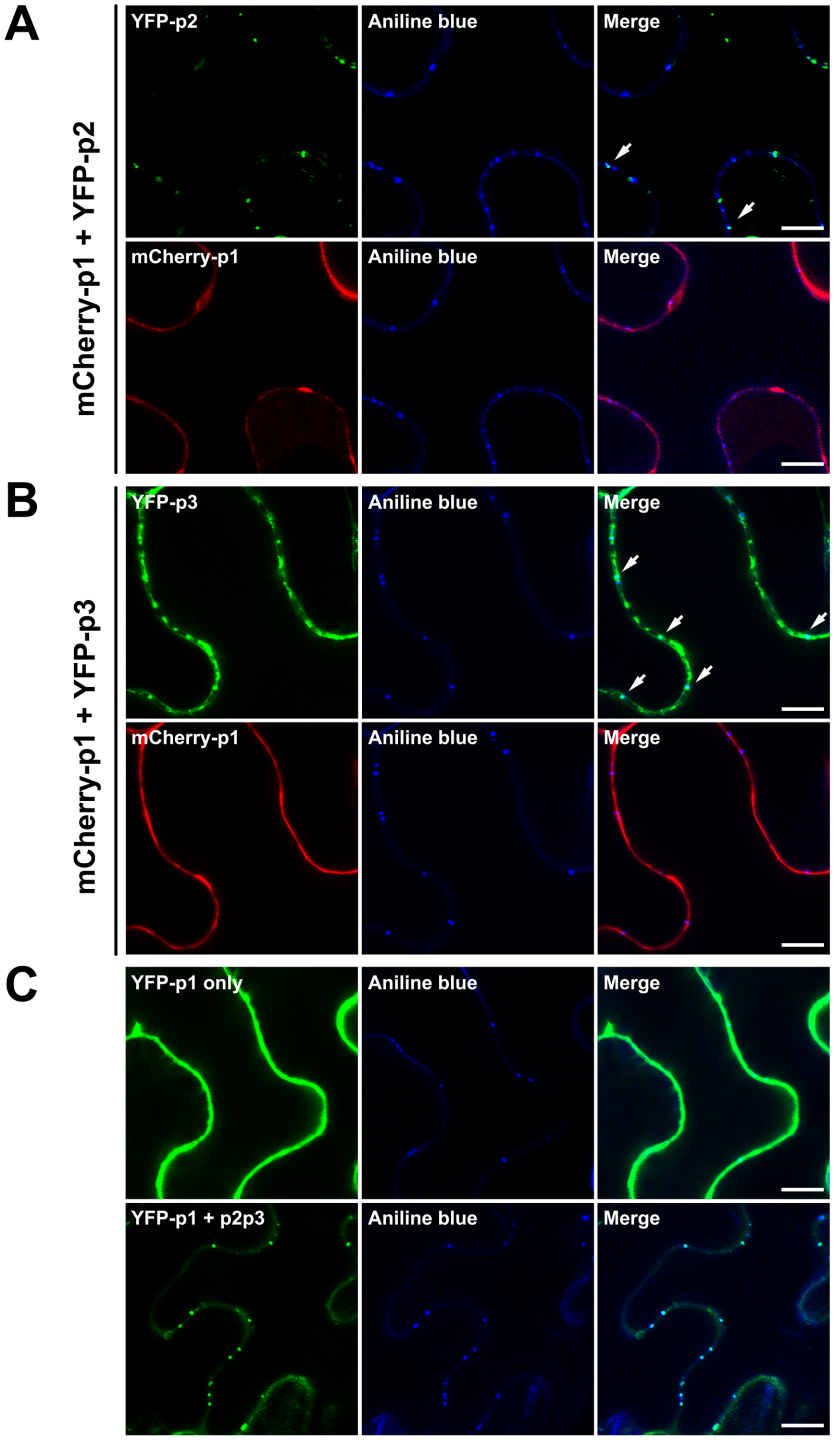
Requirement of TGBp2 and TGBp3 for efficient PD-targeting of TGBp1. (**A**) PD-targeting of TGBp1 in the presence of TGBp2. mCherry:TGBp1 and YFP:TGBp2 were co-expressed in leaf epidermal cells of *N. benthamiana* through agro-infiltration. The PD of cell periphery was visualized by aniline blue fluorochrome staining. In the most right-hand upper panel, the arrows indicate YFP:TGBp2 co-localized with PD. (**B**) PD-targeting of TGBp1 in the presence of TGBp3. mCherry:TGBp1 and YFP:TGBp3 were co-expressed in leaf epidermal cells of *N. benthamiana* through agro-infiltration. In the most right-hand upper panel, the arrows indicate the YFP:TGBp3 co-localized with PD. (**C**) PD-targeting of TGBp1 in the presence of both TGBp2 and TGBp3. Upper panels, the YFP:TGBp1 expressed alone in leaf epidermal cells. Lower panels, the YFP:TGBp1, non-fused TGBp2 and TGBp3 co-expressed in leaf epidermal cells. All confocal images were taken from a single optical plane. Bars, 10 µm.

To realize the biological significance of TGBp1 association with the stable complex of TGBp2-TGBp3-virion, we analyzed whether targeting of the potex-like TGBp1 to PD requires assistance from TGBp3 or from both TGBp2 and TGBp3 as reported for hordei-like viruses [42]–[44]. To this end, agro-compatible plasmid, expressing TGBp1, TGBp2 or TGBp3:HA with a fluorescence-protein (FP) fusion at the N-terminus of each protein, was constructed and used to analyze the TGBp2- and/or TGBp3-dependence of PD-localization of TGBp1 in leaf epidermal cells accompanied with the use of a callose-specific staining dye, aniline blue fluorochrome. The PD-localization of TGBp1 under the co-expression of either TGBp2 or TGBp3 is shown in Figures 8A and 8B. A portion of TGBp2 was localized at the PD (Figure 8A, upper panels). Similar phenomenon was observed for TGBp3; it was either localized at the PD or distributed over the cell periphery (Figure 8B, upper panels). However, TGBp1 was unable to be targeted to the PD while only TGBp2 or TGBp3 was co-expressed. The PD-localization of TGBp1 under the co-expression of both TGBp2 and TGBp3 is shown in Figure 8C. Clearly, TGBp1 was redistributed from cytosol (upper panels) to the PD (lower panels). Thus, both TGBp2 and TGBp3 are essential for efficient targeting of TGBp1 to the PD in potexvirus and the C-terminal HA-tag on TGBp3 does not interfere with TGBp1 recruitment.

### The two conserved cysteine residues, Cys-31 and Cys-46, of TGBp3 play a key role in virus cell-to-cell movement by enhancing the TGBp2- and TGBp3-dependent PD localization of TGBp1

**Figure 9:**
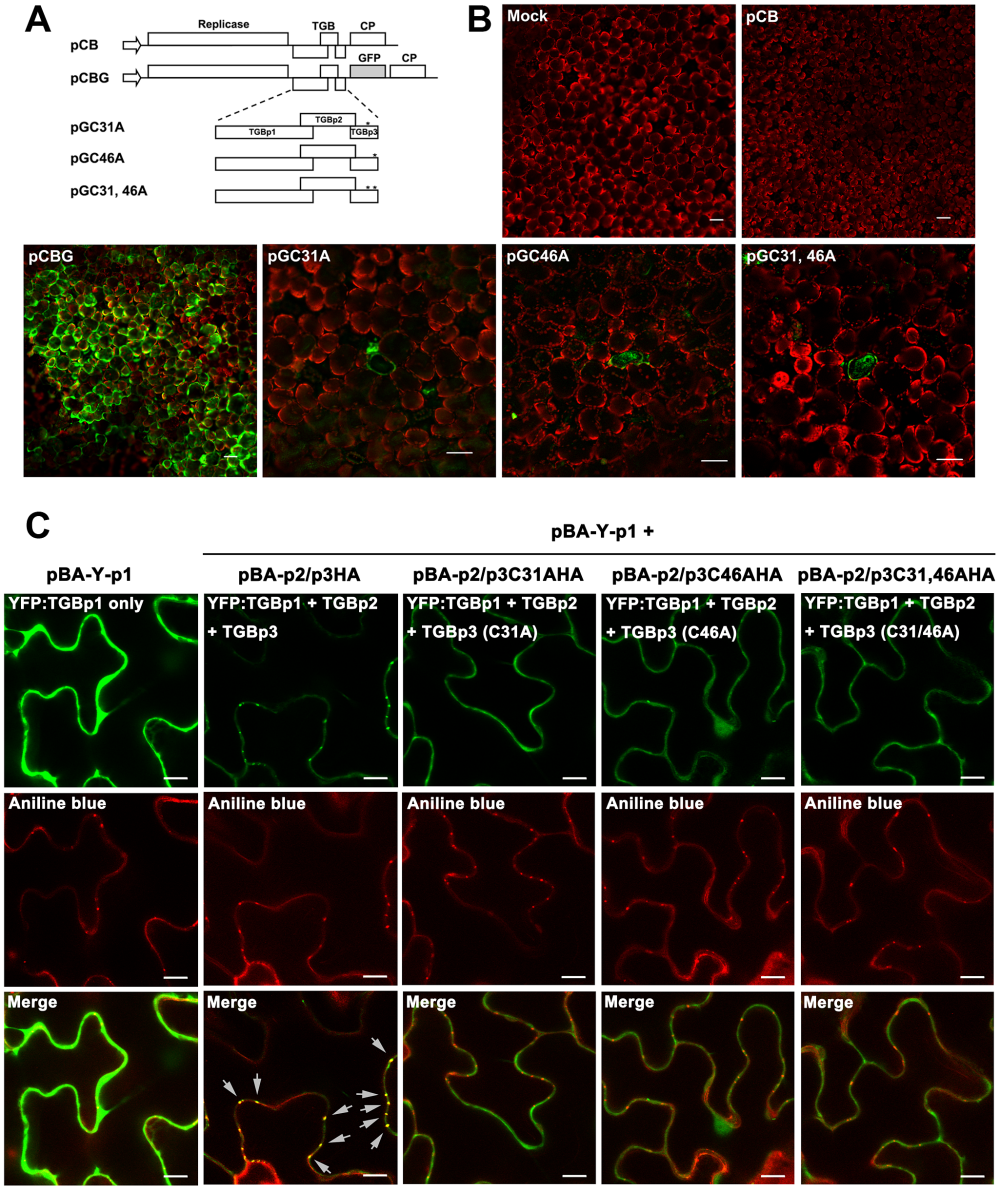
The effects of Cys-to-Ala substitutions in TGBp3 on virus cell-to-cell movement and TGBp2- and TGBp3-dependent PD targeting of TGBp1. (**A**) Diagrammatic representation of the infectious plasmid clones of wild-type (WT) and mutant BaMV. Each plasmid clone of BaMV contains a full-length BaMV genome inserted downstream the *Cauliflower mosaic virus* (CaMV) 35S promoter (as indicated by arrow). pCBG is the WT plasmid clone of BaMV derived from pCB; it has a green fluorescence protein (GFP) gene inserted between the coding sequences of TGBp3 and capsid protein (CP). pGC31A, pGC46A and PGC31, 46A are the three mutant BaMV with either one or both of the conserved cysteine residues (Cys-31 and Cys-46 are indicated by star) in the C-terminal tail of TGBp3 being replaced with alanine. (**B**) Effects of Cys-to-Ala substitutions in TGBp3 on cell-to-cell movement of BaMV. The fluorescence image of mesophyll cells of *C. quinoa* leaves inoculated with the WT plasmid clone of BaMV (pCB or pCBG) or either one of the mutant clones of BaMV ( pGC31A, pGC46A, and pGC31, 46A) was taken at 10 dpi. The white bar shown in bottom right corner of each panel is equal to 50 µm. (**C**) Effects of the Cys-to-Ala substitutions in TGBp3 on TGBp2- and TGBp3-dependent PD targeting of TGBp1. Co-expression of TGBp2 and either the WT or mutant TGBp3:HA with YFP:TGBp1 was conducted by introducing the plasmid (pBA-p2/p3HA, pBA-p2/P3C31AHA, pBA-p2/p3C46AHA or pBA-p2/p3C31, 46AHA) which expresses TGBp2 as well as the WT or mutant TGBp3:HA, and the plasmid (pBA-Y-p1) which expresses YFP:TGBp1 into *N. benthamiana* by agroinfiltration. The arrows point to the co-localized YFP:TGBp1 and callose in the PD. The white bar in the bottom right corner of each panel indicates the length of 10 µm.

To have a more direct link of the TGBp3-TGBp2-virion complex to virus movement, the effects of mutations of TGBp2 and TGBp3 on virus movement have to be examined. The Cys-to-Ala substitutions in BaMV TGBp2 have been reported to render the cell-to-cell movement of BaMV relatively inefficient and systemic movement severely inhibited [25]. To see the importance of TGBp3 to virus movement, three mutant BaMV having either one or both of the two conserved cysteine residues (Cys-31 and Cys-46) being replaced with alanine were constructed by polymerase chain reaction (PCR) using the wild-type (WT) plasmid clone of BaMV, pCBG, as template (Figure 9A). Our results showed that all of the three mutant BaMV lost their infectivity (Figure S3) and that the loss of infectivity was neither due to the defect of mutant BaMV in replication (Figure S4A) nor to the defect of TGBp3 expression (Figure S4B). Since the WT and mutant BaMV all contain an expressible green fluorescent protein (GFP) gene in their genome (Figure 9A), green fluorescence spread in mesophyll cells was expected if the tested BaMV has the ability move from cell to cell. As shown in Figure 9B, green fluorescence spread was observed in mesophyll cells of *C. quinoa* leaves 10 dpi with the WT (pCBG); however, it was restricted in a single cell as the leaves were inoculated with pGC31A, pGC46A or pGC31, 46A. These results clearly demonstrated that the two conserved cysteine residues, Cys-31 and Cys-46, of TGBp3 are essential for cell-to-cell movement of BaMV.

To clarify whether Cys-31 and Cys-46 of TGBp3 are critical for the TGBp2- and TGBp3-dependent PD targeting of TGBp1, pBA-p2/p3HA, pBA-p2/p3C31AHA, pBA-p2/p3C46AHA or pBA-p2/p3C31, 46AHA (capable of co-expressing TGBp2 with the WT or mutant TGBp3) along with pBA-Y-p1 (capable of expressing YFP:TGBp1) were introduced into *N. benthamiana* leaves by agroinfiltration. Localization of TGBp1 to the PD was analyzed two days later by examining the fluorescence of YFP:TGBp1 and aniline blue in the PD. As shown in Fig. 9C, clear co-localization of YFP:TGBp1 and aniline blue-stained callose in the PD was observed as TGBp2 and the WT TGBp3 were co-expressed; however, such a phenomenon was absent when any one of the Cys-to-Ala substitution mutant of TGBp3 was co-expressed instead. Clearly, Cys-31 and Cys-46 of TGBp3 are both critical for efficient TGBp2- and TGBp3-dependent PD targeting of TGBp1. Taken together, our results suggested that TGBp3 in the TGBp3-TGBp2-virion complex plays a key role in virus cell-to-cell movement by enhancing the TGBp2- and TGBp3-dependent PD targeting of TGBp1.

## Discussion

We have found that the TGBp2-TGBp3-based complex is able to form a stable minimal complex with virion (or that TGBp2-TGBp3-virion is the main frame of virus movement complex). Thus, the virion can be an entity for intracellular delivery of potexvirus to the PD. In addition, we have also found that TGBp3 plays a key role in virus cell-to-cell movement by enhancing the TGBp2- and TGBp3-dependent PD localization of TGBp1.

The exposure of the C-terminal tail of TGBp3 to the outer surface of ER membrane as determined by using the right-side-out membrane sample prepared from the BaMV-infected tissues (Figures 2B) and *in vitro* reconstituted TGBp3-containing proteoliposomes (Figures 2D and 2E) is consistent with the observation that the BaMV GFP:TGBp3 expressed ectopically in yeast was resistant to protease, which suggests that the N-terminal region of TGBp3 is inside the ER lumen [27]. Owing to the significant difference in the numbers of amino acid residues residing in ER lumen and cytosol (3 to 39 as shown in Figure 1A) in TGBp3, it is reasonable to predict that certain amino acid residues in the C-terminal tail of TGBp3 play a cortical ER-targeting role. This notion can be supported by the fact that I33A, I35A, I40A, I42A, and G44A mutations in the C-terminal tail eliminate the sorting of TGBp3 to cortical ER tubules [28] and that mutations of the conserved cysteine residues in the same C-terminal tail of TGBp3 cause a movement-defective phenotype of BaMV (Figure 9B). Thus, the abrogation of TGBp3 activity in intracellular trafficking and virus movement by C-terminal fusion of TGBp3 with GFP (this study), 3×HA [27], mCherry (this study) or Flag tag (this study) must be due to blocking of the functional amino acid residues or destabilization of the functional structure in the C-terminal region of TGBp3.

The strong enhancement of PD localization of TGBp1 by TGBp2 and TGBp3 as reported in this study (Figure 8) is intriguing and significant. However, the inability of TGBp2 or TGBp3 alone to enhance PD localization of TGBp1 (Figures 8A and 8B, lower panels) [7], [45] seems to indicate that TGBp2 and TGBp3 need assistance from each other for the PD localization of TGBp1 (Figure 8C, lower panel). On the basis of the results of this study and those from recent publications [27], [28], we conclude that TGBp2 in the TGBp2-TGBp3-containing membrane complex may serve as a “cooperator” that interact with TGBp1; while TGBp3 in the same complex serves as a “driver” to target the whole movement complex to the cortical ER of cell periphery. Moreover, there seems to be an important yet unknown communication mechanism between the TGBp3 “driver” and the TGBp2 “cooperator” for efficient PD localization of TGBp1 according to the observation of a significant reduction in PD localization of TGBp1 by the Cys-to-Ala substitution(s) in the C-terminal tail of TGBp3 (Figure 9C).

The existence of a minimal stable TGBp2-TGBp3-virion complex (or possibly a main frame of virus movement complex) and the absence of TGBp1 from this complex under a certain circumstance (Figures 5 and 6) suggests that the association of TGBp1 with TGBp2-TGBp3-virion is transient. In other words, the protein components in the TGBp2-TGBp3-based virus movement complex may be modified during the movement process. This feature may endow the virus being delivered a flexibility to regulate not only the efficiency of virus transport but also the cellular events concerning virus multiplication, like silencing suppression or virus disassembly [16], [18], [46], [47]. We assume that the association of TGBp1 with the complex of TGBp2-TGBp3-virion occurs after recognition of the virion by the TGBp2-TGBp3-containing membrane complex and that this association would enhance the efficiency of TGBp1 targeting to the PD (Figure 9) where an increase in size exclusion limit of PD by both TGBp1 [13]–[15] and TGBp2 [48], [49] is absolutely required for virus transport across the PD. Following the same logic of modification, the association of replicase with TGBp3-containing membrane complex (Figure 4F) may indicate the existence of a state in which certain replicase-dependent event occurs during virus movement. This idea can be supported by the finding that interaction between viral CP and the helicase-like domain of replicase is essential for virus movement [41]. The overlapping function of replicase in virus movement and replication, the presence of TGBp3 and replicase in ER at the same location [26], and the co-localization of TGBp3-containing granular vesicles with virions in X-body, where virus replication occurs [50] suggest that the TGBp3-containing membrane complex may also be a base for virus replication. Moreover, the existence of a stable TGBp3-TGBp2-virion complex (Figures 6 and 7) in combination with the strong requirement of TGBp2 and TGBp3 for efficient CP targeting to cell periphery [28], the strong enhancement of PD localization of TGBp1 by both TGBp2 and TGBp3 (Figure 8) and the defect of cell-to-cell movement caused by the Cys-to-Ala substitutions of TGBp3 (Figure 9) suggest that the PD localization of virions [51] and the subsequent virus penetration into the adjacent cell can be achieved through the help of the minimal stable TGBp3-TGBp2-virion complex in accompany with TGBp1.

**Figure 10:**
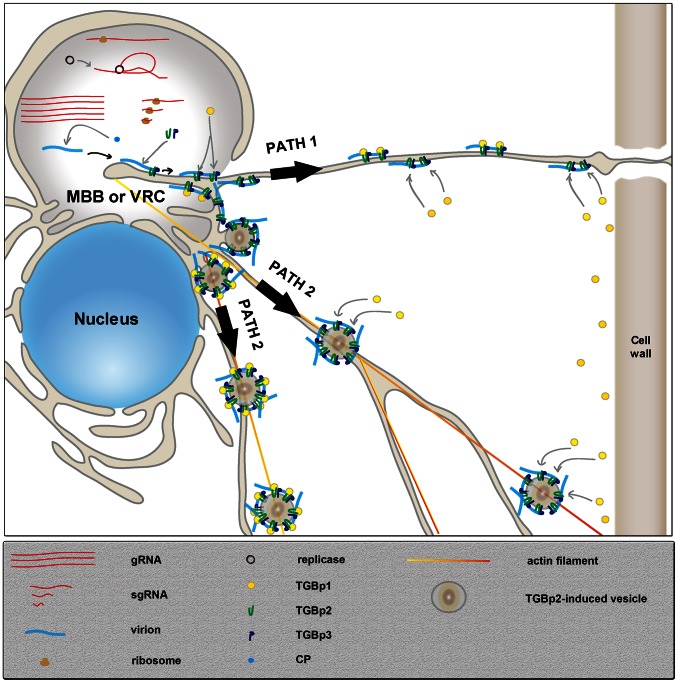
Schematic representation of the refined model for PD targeting of virions involving the formation of a stable TGBp2-TGBp3-virion complex. Two pathways were proposed for the transfer of virions from perinuclear ER-derived membrane-bound body (MBB) or viral replication complex (VRC) alongside the ER network or actin filament toward the PD. Please see text of Discussion for details.

Based on our results, a refined model for the PD-targeting of virion involving the formation of a stable TGBp2-TGBp3-virion complex is proposed (Figure 10). On the perinuclear ER-derived membrane-bound body (MBB) [29] or viral replication complex (VRC) [50], [52], [53], the putative factory for viral RNA translation, synthesis and encapsidation, the newly formed virions would associate stably with the membrane complex containing TGBp2 and TGBp3, co-expressed on the same MBB or VRC, through the interaction between TGBp2 and virion CP [28]. Subsequently, TGBp1, which is also synthesized on MBB or VRC and stays in cytosol would be recruited to the complex of TGBp3-TGBp2-virion during the transport of this complex via the interactions between TGBp1 and TGBp2, or/and TGBp1 and virion CP [28]. The TGBp1-TGBp2-TGBp3-virion complex then moved alongside the ER network toward the PD by the targeting signal of TGBp3 (Path 1 of Figure 10). Alternatively, the virus movement may adapt “Path 2”, in which the virions would associate with the TGBp2-induced ER-derived TGBp2- and TGBp3-containing membrane vesicles [20], [21]. The virion-associated vesicles are then transported to the PD through actin filament. If TGBp1 do not joint the TGBp2-TGBp3-virion movement complex on MBB or VRC, the association of cytoplasmic TGBp1 with the virion-associated complex may occur during the process of movement complex trafficking.

## Materials and Methods

### Construction of infectious BaMV clone with a tag at the C-terminal end of TGBp3

The infectious BaMV clone, pCB-P3HA, expressing the TGBp3 with an HA tag at the C-terminal end was derived from the plasmid, pCB [11]. To construct pCB-P3HA, a two-step procedure was used. The first step was to introduce an *Nhe*I site into the upstream end of the TGBp3 stop codon. In this step, the DNA fragment encompassing the stop codon of TGBp3 and the poly-A tail of BaMV genome was amplified by the forward primer (5′-TGCATGCATTGCACTAGGCTAGCTAGGGTTTGTTAAGTTTCCTTC -3′) containing *Nsi*I and *Nhe*I sites (the underlined bases, respectively) and the reverse primer (5′-GCGTACCGAATTCGAGCTCTTTTT-3′) containing *Sac*I site (the underlined bases) using pCB as template. Afterwards, the amplified DNA was digested with *Nsi*I (located 21 bases downstream from the start codon of TGBp3) and *Sac*I (located at the end of poly-A tail) and used to replace the coding sequence for TGBp3 on the pCB plasmid after digestion with the same restriction enzymes to generate the pCB-B+ plasmid. The second step was to introduce an HA tag into the downstream region of the TGBp3 coding sequence. In this step, the DNA sequence encoding TGBp3:HA was amplified by the forward primer (5′-CAACCCTTTTCCTCATCACCAG-3′) and the reverse primer (5′-TGCACTAGGCTAGCGGCGTAGTCGGGCACGTCGTAGGGGTAGCTGGAGGTGGTGTGGTAGC-3′) containing the DNA sequences of HA-tag and *Nhe*I site (the underlined bases). After *Nsi*I and *Nhe*I digestion, the DNA fragment was cloned into the compatible sites on pCB-B+ to generate the pCB-P3HA plasmid. The other infectious BaMV clone, pCB-P3F, with a Flag tag at the C-terminal end of TGBp3 was constructed using the same procedure, except for the design of a tag sequence in the reverse primer.

### Construction of plasmid transiently expressing YFP:TGBp2, YFP:TGBp3:HA, YFP:TGBp1, mCherry:TGBp1,TGBp2 or TGBp3:HA in *N. benthamiana*

To construct pBA-Y-p2 which expresses YFP-TGBp2 in *N. benthamiana*, the DNA encoding TGBp2 was first amplified from the template, pCBG, using the two primers, KFP2 (5′-CGGCGGGGTACCGGGACCAGCCTCTTCATCTG-3′) and SRP2 (5′-TCCTCCCCCGGGTTAGCATGGTGGGTGATTCCG-3′) containing *Kpn*I and *Xma*I sites (the underlined bases in each primer), respectively. After digestion with *Kpn*I and *Xma*I, the DNA was cloned into the compatible sites of the pWEN25 plasmid [54], [55] to generate pW25-p2, with which a YFP:TGBp2 fusion can be obtained. The YFP:TGBp2-encoding DNA fragment on pW25-p2 was cut out by using *Xho*I and *Sac*I, and cloned into the compatible sites of pBA002 [54], [56] to generate pBA-Y-p2.

To construct pBA-Y-p3HA expressing YFP:TGBp3:HA in *N. benthamiana*, the DNA encoding TGBp3 was first amplified from pCB-P3HA using the two primers, 5′-TGBp3-KpnI (5′-CGGCGGGGTACCGGCTAAACACTGACACACTATG-3′) and 3′-TGBp3-XmaI (5′-TCCTCCCCCGGGTCAGGCGTAGTCGGGCACG-3′), which contain *KpnI* and *Xma*I sites (the underlined bases in each primer), respectively. The amplified DNA was then digested with *Kpn*I and *Xma*I, and cloned into the compatible sites of pWEN25 to generate pW25-p3HA. The DNA fragment encoding YFP:TGBp3:HA was then cut out from pW25-p3HA using *Xho*I and *Sac*I, and cloned into the compatible sites of pBA002 to generate pBA-Y-p3HA.

To construct pBA-Y-p1 transiently expressing YFP:TGBp1 in *N. benthamiana*, a two-step procedure was adopted. In the first step, the two primers, pBA-nXFP(F) (5′-TAGAGGATCTCGAGATGGTGAGCAAGGGCGAGG-3′) and pBA-nXFP(R) (5′-CAGGCCTACGCGTCTTGTACAGCTCGTCCATGC-3′), containing *Xho*I and *Mlu*I sites (the underlined bases in each primer), respectively, were used to amplify the DNA encoding YFP using pWEN25 as template. After digestion of the amplified YFP DNA with *Xho*I and *Mlu*I, the DNA fragment was cloned into the compatible sites of pBA002 to generate pBA-YFP. In the second step, the TGBp1-encoding DNA was amplified from the pCBG plasmid using the two primers, pBA-nXFP-P1(F) (5′-CAGGCCTACGCGTATGGATAACCGGATAACTGACC-3′) and pBA-nXFP-P1(R) (5′-TCGAGCTCACTAGTTCAGGTGGTCTGGCCAGATG-3′), containing *Mlu*I and *Spe*I sites (the underlined bases in each primer), respectively. The TGBp1 DNA was then digested with *Mlu*I and *Spe*I and cloned into the compatible sites of the pBA-YFP plasmid to generate pBA-Y-p1.

To construct pBA-p2/p3HA transiently co-expressing TGBp2 and TGBp3:HA in *N. benthamiana*, the two primers, pBA-nXFP-P2(F) (5′- CAGGCCTACGCGTATGGACCAGCCTCTTCATCTG -3′) and pBA-nXFP-P3(R) (5′- TCGAGCTCACTAGTCTAGGCGTAGTCGGGCACGTC -3′), containing *Mlu*I and *Spe*I sites (the underlined base in each primer), respectively, were used to amplify the DNA encoding TGBp2/TGBp3HA using pCB:P3HA as template. The amplified DNA was then digested with *Mlu*I and *Spe*I, and cloned into pBA002 to generate pBA-p2/p3HA.

To construct pBA-mCh-p1 which transiently expresses mCherry:TGBp1 in *N. benthamiana*, a two-step procedure was adopted. In the first step, the mCherry:TGBp3 DNA fragment in p35S-mCherry-TGBp3 [28] plasmid was cut out by *Xba*I and *Xho*I, and cloned into the compatible sites of pBA002 to generate pBA-mCh-p3. In the second step, the TGBp1 DNA fragments were amplified from pCB using the two primers, pBA-mCh-P1(F) (5′-CGCGGATCCCCCGGGATGGAT AACCGGATAACTGACC-3′) and pBA-nXFP-P1(R) (5′-TCGAGCTCACTAGTTCAGGxTGGTCTGGCCAGATG-3′), containing *Xma*I and *Spe*I sites (the underlined bases in each primer), respectively. The TGBp1 DNA was then used to replace the TGBp3 DNA on the pBA-mCh-p3 plasmid after digestion of both the TGBp1 DNA and pBA-mCh-p3 plasmid DNA with *Xma*I and *Spe*I. The resultant plasmid was designated as pBA-mCh-p1.

### Construction of infectious plasmid clones of BaMV with Cys-to-Ala substitutions in TGBp3

Mutant BaMV with Cys-to-Ala substitution(s) at positions 31 and/or 46 of TGBp3 were constructed using a Site-Directed Mutagenesis Kit (Stratagene). The pCBG plasmid [57], which contains the full-length genome of WT BaMV was used as template for mutagenesis. The primers, C31Af (5′-CAACAGCATCTGCCCCCACCAGCAGAAATAATAATAAACGGGC-3′), C31Ar (5′-GCCCGTTTATTATTATTTCTGCTGGTGGGGGCAGATGCTGTTG-3′), C46Af (5′-CTATATCCATTAGGGGCAACGCATACCACACCACCTCCAGC-3′) and C46Ar (5′-GCTGGAGGTGGTGTGGTATGCGTTGCCCCTAATGGATATAG-3′), are used for the construction of mutant BaMV by polymerase chain reaction (PCR). The three infectious plasmid clones of mutant BaMV finally obtained were named pGC31A, pGC46A and pGC31, 46A.

### Construction of satBaMV-derived plasmid which expresses WT or mutant TGBp3:HA

To construct satBaMV-derived plasmid which expresses WT or mutant TGBp3:HA, we first synthesized the DNA coding for TGBp3:HA by PCR using pCB-P3HA as template, and FP3-*Bst*XI (5′-CTGCAGAACCAAGACGATGGAATCACCCACCATGCTAAAC-3′) and RHA-*Eco*NI (5′-CTGCAGCCTCTGGGAGGTCAGGCGTAGTCGGGCACGTC -3′) as primers. The PCR product was digested with *Bst*XI and *Eco*NI and used to replace the DNA coding for the P20 protein of satellite BaMV by cloning into the compatible sites of pCBSF4 [5]. The resultant plasmid was named p3HA. The initiation codon (ATG) of the P20 protein, which remains on p3HA and locates just upstream of the coding sequence of TGBp3:HA, was mutated into GTG through site-directed mutagenesis using FA160G (5′- GACGCTTACCAAGACGGTGGAATCACCCACC-3′) and RA160G (5′- GGTGGGTGATTCCACCGTCTTGGTAAGCGTC -3′) as primers. The resultant plasmid was used as template for mutagenesis of Cys-31 and/or Cys-46 of TGBp3. The primers used for Cys-31-Ala substitution are C31Af and C31Ar; they are C46Af and C46Ar for Cys-46-Ala substitution. The plasmids finally obtained were named p3C31AHA, p3C46AHA and p3C31, 46AHA.

### Construction of plasmid clone of BaMV which expresses mutant TGBp3:HA

To construct plasmid clone of BaMV which expresses mutant TGBp3 with an HA fusion at the C-terminal end, the DNA fragments coding for each of the three Cys-to-Ala substitution mutants of TGBp3:HA were amplified from the plasmid, p3C31AHA, p3C46AHA or p3C31, 46AHA by the two primers, FP3-KpnI (5′- CGGCGGGGTACCGGCTAAACACTGACACACTATG-3′) and RP3HA-NheI (5′- TGCACTAGGCTAGCGGCGTAGTCGGGCACGTCGTAGGGGTAGCTGGAGGTGGTGTGGTAGC -3′), digested with *Nhe*I and *Nsi*I and used to replace the corresponding WT TGBp3:HA DNA fragment on pCB-P3HA. The resultant plasmids were named pCB-p3C31AHA, pCB-p3C46AHA and pCB-p3C31, 46AHA.

#### Construction of plasmid co-expressing TGBp2 and either WT or mutant TGBp3:HA in *N. benthamiana*

To construct plasmids which co-express TGBp2 and either the WT or mutant TGBp3:HA , we first synthesized the DNA coding for TGBp2 and either the WT or mutant TGBp3 by PCR using pCB-p3HA, pCB-p3C31AHA, pCB-p3C46AHA or pCB-p3C31, 46AHA as template. The forward and reverse primers used for the synthesis are FP2-MluI (5′-CAGGCCTACGCGTATGGACCAGCCTCTTCATCTG-3′) and RHA-SpeI (5′-TCGAGCTCACTAGTCTAGGCGTAGTCGGGCACGTC-3′). After digestion of the PCR DNA with *Mlu*I and *Spe*I, the DNA fragments were cloned into the compatible sites of pBA002. The resultant plasmids were named pBA-p2/p3HA, pBA-p2/p3C31AHA, pBA-p2/p3C46AHA and pBA-p2/p3C31, 46AHA, respectively.

### Fractionation of the BaMV-infected tissues

The leaves of *N. benthamiana* were inoculated with pCB, pCBG [11] or pCB-P3HA and the infected systemic leaves were collected 21–28 days later. The healthy or infected leaf sample (5 g) was ground with liquid nitrogen. Then, 20 ml of 4°C-cold extraction buffer (50 mM imidazole, pH 7.0, 250 mM sucrose, 10% glycerol, 50 mM EDTA) supplemented with 170 µl protease inhibitor cocktail (Sigma), 1% (v/v) β-mercaptoethanol (βME), 5 mM each of DTT and 6-aminocaproic acid (EACA), and 1 mM PMSF, was added into the ground sample for further homogenization with a disperser. The homogenate was filtered through a 4-layer miracloth and centrifuged at 1,000 ***g***, 4°C for 10 min to collect the pellet (P1) and supernatant (S1). The S1 fraction was further centrifuged at 30,000 ***g***, 4°C for 30 min to separate the cytosolic (S30) and membrane (P30) fractions.

### The association of TGBp3:HA with endoplasmic reticulum (ER)

Method used for examining the co-fractionation of TGBp3:HA with ER protein markers, TGBp2 and BiP, is similar to that reported previously [19]. Briefly, the P30 sample prepared from 5 g of BaMV-infected tissues was resuspended with 1 ml of buffer (50 mM imidazole, pH 7.0) containing 20% sucrose and subjected to centrifugation in a 34 and 45% step sucrose gradient. The membrane sample in the interface of 34 and 45% sucrose was recovered and diluted with two volumes of the same imidazole buffer without sucrose and glycerol. Then, the membrane sample was layered on a 20 to 45% linear sucrose density gradient and centrifuged at 143,000 ***g***, 4°C for 4 h. The fractionated samples from top to bottom were collected (0.6 ml/fraction; totally 18 fractions) and then subjected to western blot analysis using anti-HA, anti-BiP (Santa Cruz Biotechnology), and polyclonal anti-TGBp2.

### Multimerization of TGBp3:HA in the membrane fraction, P30

Cu(OP)_2_ was prepared in fresh by mixing CuSO_4_ with *o*-phenanthroline in a molar ratio of 1∶2 in the grinding buffer (50 mM imidazole, pH 7.0, 250 mM sucrose) as described [58]. P30 sample from 0.8 g healthy or pCB-P3HA-infected leaves was pelleted at 30,000 ***g***, fully resuspended with grinding buffer in a micro test tube by gentle agitation. Then, the sample was centrifuged and fully resuspended again to wash out the residual reductants and EDTA. The resuspended P30 was equally divided into 4 microcentrifuge tubes (0.2 g per tube). Two of them were incubated with and two without 0.5 mM Cu(OP)_2_ at 4°C for 1 h on a rotation mixer. The oxidation reaction was stopped by addition of *N*-ethylmaleimide and EDTA both at a final concentration of 5 mM, and incubated in the dark for 10 min. Multimerization of TGBp3:HA was also examined using the membrane-impermeable bismaleimide crosslinker, BM(PEG)_2_. The crosslinking reaction was performed using a procedure similar to that used for Cu(OP)_2_ oxidation, except that BM(PEG)_2_ was used at a concentration of 1 mM. The crosslinking reaction was allowed to proceed for 2 h at 4°C and terminated with βME at a final concentration of 70 mM.

### Chemical modification of TGBp3:H6

Initially, 0.2 µg of TGBp3:H6-containing proteoliposomes were incubated with buffer H only or with buffer H containing 1 mM 4-acetamido-4′-maleimidylstilbene-2, 2′-disulfonic acid disodium salt (AMS, Molecular Probes) for 40 min at room temperature to block the water-accessible cysteines in TGBp3:H6. Then, the excess AMS in reaction mixture was inactivated with 3 mM β-mercaptoethanol (βME) and removed by dialysis against buffer H for 1 h at 4°C. The proteoliposome samples pretreated or untreated with AMS were both divided into two aliquots. One was treated with buffer H; the other with buffer H containing 1% Triton X-100 to disrupt the proteoliposomes. The four samples were further incubated with 0.2 mM *N*_α_-(3-maleimidylpropionyl) biocytin (MPB, Molecular Probes) at room temperature for 20 min to biotinylate TGBp3:H6. The excess MPB was quenched with 1 mM βME and the biotinylated TGBp3:H6 was precipitated with acetone before Tricine SDS-PAGE and blotting analyses. Biotinylation of TGBp3:H6 was assessed using avidin-HRP (Sigma) coupled with HRP-conjugated secondary antibody; the relative content of TGBp3:H6 in reaction mixture was assessed using anti-His-tag antibody coupled with HRP-conjugated secondary antibody.

### Extraction and immunoprecipitation of the TGBp3-based protein complex

To solubilize the TGBp3-based protein complex, the P30 sample was fully resuspended and washed with 5 ml of extraction buffer twice to remove residual reducing agent in the P30. The washed P30 was again fully resuspended in 1 ml of extraction buffer containing 1% (w/v) sarkosyl and incubated at 4°C for 2 h by a rotamixer (ELMI Rotamix RM1). After incubation, the sample was centrifuged at 100,000 ***g***, 4°C for 30 min to collect the pellet (P100) and the supernatant (S100).

Prior to immunoprecipitation of the TGBp3-based protein complex, 1 ml of S100 was incubated with 0.1 ml of 50% (v/v) Protein A-Sepharose CL-4B suspension (designated as PA) for 1.5 h, and centrifuged at 3,000 ***g***, 4°C for 2 min to remove protein components interacting non-specifically with PA. The S100 pre-cleaned with PA was designated as input (Inp). To immunoprecipitate the TGBp3-based protein complex, 0.5 ml of Inp was mixed with 10 µl of anti-HA (0.5 mg/ml) on a rotamixer for 1 h and incubated with 20 µl of 50% PA for 12 h. After incubation, the sample was centrifuged at 3,000 ***g***, 4°C for 2 min to separate the supernatant (IpS) and pellet (IpP). The IpP was washed once with 1 ml of extraction buffer containing 0.5% Sarkosy and twice with 1 ml of grinding buffer (50 mM imidazole, pH 7.0, 250 mM sucrose, 3 mM EDTA) by centrifugation at 3,000 ***g***, 4°C for 2 min. To avoid the loss of IpP, about 20–100 µl of buffer was left over at each wash step. The final IpP was stored at −80°C. For western blot analyses, the IpP was dissolved with sample application buffer, boiled at 95°C for 10 min, subjected to Tricine SDS-PAGE, transferred to PVDF membrane and probed with a specific antibody.

### Delipidation of the TGBp3-containing membrane protein sample

To remove phospholipids or detergent from the TGBp3-containing membrane protein sample, methanol/chloroform precipitation [59] was performed. Briefly, 100 µl of the suspended P30 or S100 was mixed sequentially with 400 µl of methanol, 200 µl of chloroform and 300 µl of deionized water. The sample was vortexed, briefly spun after each mix, and centrifuged at 13,200 ***g*** in a microcentrifuge for 1 min. After centrifugation, the upper aqueous layer was removed and 300 µl of methanol was added into the interface and bottom layer of the sample. The mixture was centrifuged again at 13,200 ***g*** for 2 min to pellet proteins. The protein pellet was dried under a stream of air before subjecting to immunological analyses.

### Chemical extraction and partial trypsin digestion of TGBp3 in membrane

Prior to chemical extraction and trypsin digestion of TGBp3 in membrane vesicles, 1 ml of grinding buffer was added into the P30 sample prepared from 5 g of leaf tissues before gentle grinding with a hand homogenizer to fully suspended. For chemical extraction, 180 µl of grinding buffer supplemented with 1 mM PMSF was added into 20 µl of the resuspended P30 (equivalent to 0.1 g leaf tissues) in an ultracentrifuge tube. The mixture was centrifuged at 30,000 ***g***, 4°C for 30 min to obtain P30 again. Then, 200 µl of grinding buffer containing 4 M urea or 1 M KCl, or 200 µl of 0.1 M Na_2_CO_3_ (pH 11) solution was added into the sample tube. The P30 in the tube was fully resuspended with gentle agitation, incubated on ice for 30 min and centrifuged at 30,000 ***g***, 4°C for 30 min to separate the supernatant (S30) and pellet (P30).

For partial trypsin digestion, 0, 10, 20, 40, 80 or 160 µg of freshly prepared trypsin was added into 40 µl of resuspended P30 (equivalent to 0.2 g leaf tissues) in a microcentrifuge tube. The final volume of each reaction was adjusted to 200 µl with ddH_2_O. The digestion reaction was carried out at 37°C for 30 min and quenched with 5 mM PMSF and 5 mM EACA. After delipidation, the digested protein sample was subjected to Western blotting.

### RNA-dependent RNA polymerase assay

The method used for endogenous RdRp assay was the same as that described previously [41]. In brief, the IpP sample prepared from S100 was resuspended and incubated with reaction buffer containing 30 mM Tris (pH 8.8), 50 mM NaCl, 20 mM DTT, 10 mM MgCl_2_, 2 mM each of ATP, CTP and GTP, 2 µM of UTP, and 100 µCi [α-^32^P] UTP at 25°C for 3 h. The RNA products were extracted with phenol/chloroform, precipitated with ethanol, electrophoresed on a 0.8% agarose gel and detected by autoradiography.

### Purification of virions

The method used for virion purification was modified from a previously published paper [60]; the whole purification process was performed at 4°C. Briefly, 15 g of the systemic leaves of *N. benthamiana* infected with pCB-P3HA and collected 28 days after inoculation was ground with 20 ml of borate buffer (0.5 M borate, pH 9.0, 1 mM EDTA, 0.5% (v/v) βME, 0.1 mM PMSF and 5 mM EACA). The leaf homogenate was filtered through a 4-layer miracloth and centrifuged at 12,000 ***g*** for 10 min to collect the supernatant. Then, K_2_HPO_4_ and CaCl_2_, both at a final conc. of 0.04 M, were added drop by drop to the supernatant. The mixture was stirred for 10 min and centrifuged at 12,000 ***g*** for 10 min. Subsequently, Triton X-100 and PEG 6000 were added into the supernatant; the final concentrations of the two chemicals were 2% and 6%, respectively. The mixture was stirred for 30 min and centrifuged at 12,000 ***g*** for 10 min. The pellet thus obtained was resuspended with 4 ml of BE buffer (0.05 M borate, pH 8.0, 1 mM EDTA) and centrifuged through a 1-ml cushion of 20% sucrose at 143,000 ***g*** for 1 h. The virions in the pellet was finally resuspended with 0.2 ml of BE buffer and stored at −20°C.

### Immunoelectron microscopy

Virions (20–40 ng CP/µl) were adsorbed onto the 200–300 mesh nickel grids pre-coated with formvar and carbon for 7 min. The grids were briefly rinsed with PBS buffer (1.47 mM NaH_2_PO_4_·H_2_O, 8.09 mM Na_2_HPO_4_, pH 7.4, 145 mM NaCl), incubated with blocking buffer (PBS buffer plus 5% BSA (w/v)) for 15 min, with PBS buffer supplemented with 5% BSA (w/v) and 5% normal donkey serum (v/v) for 15 min, and finally with PBS buffer supplemented with 1% BSA and 1% normal donkey serum (BB1) for 1 min to complete the blocking process. The grids were incubated with monoclonal anti-HA (Roche) at a dilution of 1∶40 in BB1, washed four times with PBS buffer and once with BB1 (5 min each time), and incubated with 18 nm gold-conjugated donkey anti-mouse antibody at a dilution of 1∶20 in BB1 for 1 h. For double labeling, the grids were washed thrice with PBS buffer, once with BB1 (5 min each time), and incubated with 100-fold diluted pre-cleaned TGBp2 antibody [19], 2000-fold diluted TGBp1 antibody [6] for 2 h, 100-fold diluted virion-specific CP antibody for 1 h. After incubation with a specific antibody, the grids were washed four times with PBS buffer and once with BB1 (5 min each time), incubated with 12 nm gold-conjugated donkey anti-rabbit antibody (1∶20 diluted in BB1) for 1 h, washed four times with PBS (5 min each time) and fixed with PBS buffer plus 0.25% glutaraldehyde for 10 min. Finally, the grids were washed thrice with ddH_2_O (1 min each time) and stained with 2% uranyl acetate (UA) in ddH_2_O for 2 min. The residual UA solution on the grids was gently blotted with filter paper, and the grids were dried for 3 h in a dry cabinet. All of the sample grids were analyzed by a Philips CM 100 TEM.

### Preparation of *Agrobacterium* for plant agroinfiltration

Preparation and transformation of *Agrobacterium tumefaciens* strain ABI were performed as previously described [61]. The *A. tumefaciens* harboring a designed plasmid was grown at 28°C for 48 h in a 20-ml culture tube containing 5 ml of LB medium supplemented with 50 µg/ml spectinomycin. Then, 200 µl of the culture were transferred to 10 ml of LB medium supplemented with 10 mM MES buffer, pH 5.6, 40 µM acetosyringone and 50 µg/ml spectinomycin before further grown at 28°C for 16 h in a shaker. The cells were harvested by centrifugation and resuspended in a solution containing 10 mM MgCl_2_ and 150 µM acetosyringone to a final optical density of 1.0 at 600 nm. The cell sample was incubated at room temperature for 3 h without shaking. If necessary, two suspensions of *Agrobacterium*, each harboring a specific plasmid, were mixed in a 1∶1 ratio prior to agoinfiltration. The *Agrobacterium* was infiltrated into the intercellular space of 4∼5-week-old *N. benthamiana* leaves. The plant was kept growing in a greenhouse for 48 h with 16 h light/8 h dark before being analyzed with fluorescence microscopy.

### Fluorescence microscopy

To visualize PD in leaf epidermal cells, we infiltrated aniline blue fluorochrome (Biosupplies) (0.1 mg/ml in water) into the agro-infected leaves of *N. benthamiana* and analyzed the sample immediately using an Olympus FV1000 laser scanning inverted microscope with a 60×/1.2 immersion objective lens. Image was captured by the use of FLUOVIEW software with filters for aniline blue fluorochrome (405 nm laser, BP480–510), YFP (515 nm, BP530–560), and mCherry (543 nm, BP 565–660), respectively. To obtain the images of co-expressed mCherry and YFP, the signals of mCherry and YFP were captured sequentially at the same focal plane of the same cell. All images were processed using Photoshop (Adobe).

## Supporting Information

Figure S1 **Expression and purification of TGBp3 with a His-tag fusion at the C-terminus.** (**A**) Amino acid sequence of TGBp3 with six histidine residues fused at its C-terminus. (**B**) Expression of TGBp3:H6 in *E. coli* as analyzed by western blot using anti-His tag. R, R/v and R/p3cH6 indicate the host, *E. coli* Rosetta (DE3)/pLysSRARE, the host with vector (pET21d) and the host with TGBp3:H6-expressing recombinant plasmid (p3cH6), respectively. B, B/v, and B/p3cH6 indicate the host, *E. coli* BL21(DE3), the host with vector (pET21d), and the host with TGBp3:H6-expressing recombinant plasmid (p3cH6), respectively. (**C**) Purification of TGBp3:H6 using TALON resin. The purified TGBp3;H6-containing samples were subjected to Coomassie blue staining after Tricine SDS-PAGE and western blot analysis. S, the supernatant fraction obtained by centrifugation of *E. coli* cell lysate containing overexpressed TGBp3:H6 in inclusion form. I, the 6 M Gn-HCl solubilized TGBp3:H6 sample (see Text S1 for method of preparation). F, the flowthrough sample from TALON resin. 1, 2 and 3 are three fractions of the eluate from TALON resin. M, D, Tr, and Te indicate monomeric, dimeric, trimeric and tetrameric forms of TGBp3:HA, respectively.
(TIF)

Figure S2 **Confirmation and purification of the** ***in vitro*** **reconstituted TGBp3:H6-containing proteoliposomes.** The TGBp3-containing proteoliposomes were prepared *in vitro* using a micelle-vesicle transition method. (**A**) Confirmation of successful *in vitro* reconstitution of TGBp3:H6-containing proteoliposomes by linear Ficoll gradient (1 to 10%) centrifugation. Left panel, centrifugation of liposoimes containing phospholipids only. Middle panel, centrifugation of TGBp3:H6 solubilized with buffer containing 1% Triton X-100. Right panel, centrifugation of *in vitro* reconstituted TGBp3-containing proteoliposomes. In these panels, pink triangle indicates phospholipids; blue solid circle indicates residual protein in purchased asolectin (left panel) or TGBp3 (right panel). (**B**) Confirmation of successful *in vitro* reconstitution of TGBp3:H6-containing proteoliposomes by flotation assay. Method used for flotation assay is described in Text S1. Left panel, flotation of *in vitro* reconstituted TGBp3:H6-containing proteoliposomes in 0, 25%, 30% sucrose step gradient. Middle panel, distribution of TGBp3:H6 in step sucrose gradient as analyzed by silver staining. Right panel, distributions of phospholipids and TGBp3:H6 in the step sucrose gradient. Pink triangle and blue solid circle indicate phospholipids and TGBp3:H6, respectively.
(TIF)

Figure S3 **The loss of infectivity of the mutant BaMV with Cys-to-Ala substitution(s) in TGBp3.** (**A**) Effect of Cys-to-Ala substitution in TGBp3 on the infectivity of BaMV. The fluorescence images of leaves were taken 6 dpi for *Chenopodium quinoa* (left panel) and 17 dpi for *Nicotiana benthamiana* (right panel). (**B**) Western blot analysis of CP in plant leaves. The protein samples were extracted from leaves of *C. quinoa* 10 dpi (left panel) and *N. benthamiana* 17 dpi (right panel). Protein samples equivalent to 80 µg of inoculated leaves were used for the analysis of CP. The CP sample in the left-most lane of both panels was purified from an *Escherichia coli* overexpression system.
(TIF)

Figure S4 **The mutant BaMV with Cys-to-Ala substitution(s) in TGBp3 is active in replication and TGBp3 expression.** (**A**) Northern blot analysis of viral RNA in protoplasts of *N. benthamiana*. Total nucleic acids extracted from protoplasts infected with each of the plasmid clones of BaMV were separated in a 1.2% agarose gel, blotted onto a nylon membrane, and probed with a 600-base long ^32^P-labeled RNA complementary to the 3′ end of BaMV genomic RNA. Viral RNA in the left-most lane was purified from *C. quinoa* inoculated with the infectious WT plasmid clone of BaMV, pCB. The full-length genomic RNA (gRNA) and internal control (28S ribosomal RNA [rRNA]) are shown. (**B**) Contents of the WT and mutant TGBp3:HA in *N. benthamiana* as analyzed by western blot. M and D indicate the monomeric and dimeric forms of TGBp3:HA, respectively.
(TIF)

Text S1 **Details, including** **Results** **as well as** **Materials and Methods** **, for Figures S1, S2, S3 and S4.**
(DOC)
