# Supplementary figures and images for: The Stable Association of Virion with the Triple-gene-block Protein 3-based Complex of Bamboo mosaic virus
Source: PLoS Pathog. 2013 Jun 6;9(6):e1003405. doi: 10.1371/journal.ppat.1003405 (PMC3675025; doi:10.1371/journal.ppat.1003405)

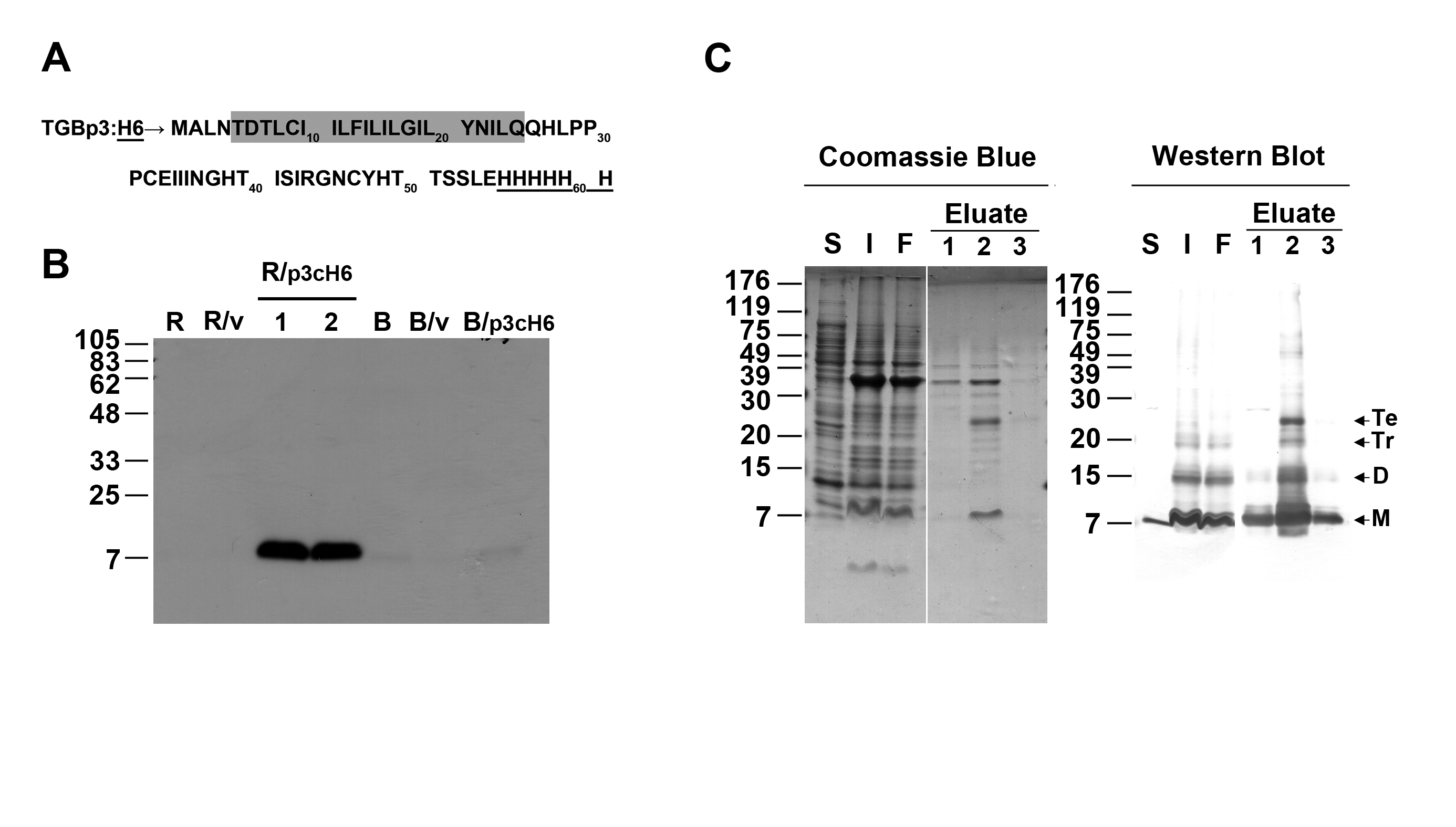

Supplement: Figure S1 — Expression and purification of TGBp3 with a His-tag fusion at the C-terminus. (A) Amino acid sequence of TGBp3 with six histidine residues fused at its C-terminus. (B) Expression of TGBp3:H6 in E. coli as analyzed by western blot using anti-His tag. R, R/v and R/p3cH6 indicate the host, E. coli Rosetta (DE3)/pLysSRARE, the host with vector (pET21d) and the host with TGBp3:H6-expressing recombinant plasmid (p3cH6), respectively. B, B/v, and B/p3cH6 indicate the host, E. coli BL21(DE3), the host with vector (pET21d), and the host with TGBp3:H6-expressing recombinant plasmid (p3cH6), respectively. (C) Purification of TGBp3:H6 using TALON resin. The purified TGBp3;H6-containing samples were subjected to Coomassie blue staining after Tricine SDS-PAGE and western blot analysis. S, the supernatant fraction obtained by centrifugation of E. coli cell lysate containing overexpressed TGBp3:H6 in inclusion form. I, the 6 M Gn-HCl solubilized TGBp3:H6 sample (see Text S1 for method of preparation). F, the flowthrough sample from TALON resin. 1, 2 and 3 are three fractions of the eluate from TALON resin. M, D, Tr, and Te indicate monomeric, dimeric, trimeric and tetrameric forms of TGBp3:HA, respectively. (TIF) [file ppat.1003405.s001.tif]

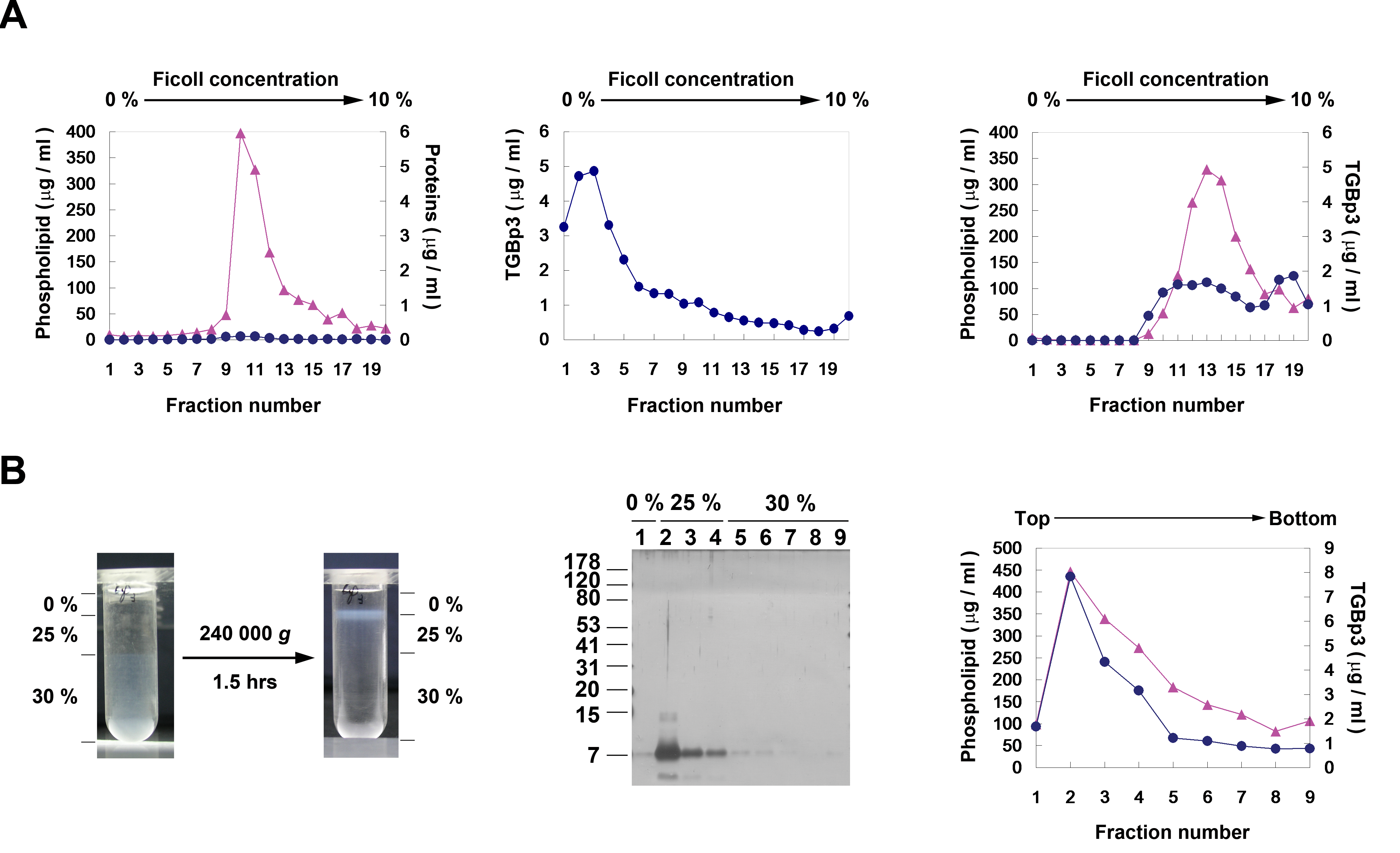

Supplement: Figure S2 — Confirmation and purification of the in vitro reconstituted TGBp3:H6-containing proteoliposomes. The TGBp3-containing proteoliposomes were prepared in vitro using a micelle-vesicle transition method. (A) Confirmation of successful in vitro reconstitution of TGBp3:H6-containing proteoliposomes by linear Ficoll gradient (1 to 10%) centrifugation. Left panel, centrifugation of liposoimes containing phospholipids only. Middle panel, centrifugation of TGBp3:H6 solubilized with buffer containing 1% Triton X-100. Right panel, centrifugation of in vitro reconstituted TGBp3-containing proteoliposomes. In these panels, pink triangle indicates phospholipids; blue solid circle indicates residual protein in purchased asolectin (left panel) or TGBp3 (right panel). (B) Confirmation of successful in vitro reconstitution of TGBp3:H6-containing proteoliposomes by flotation assay. Method used for flotation assay is described in Text S1. Left panel, flotation of in vitro reconstituted TGBp3:H6-containing proteoliposomes in 0, 25%, 30% sucrose step gradient. Middle panel, distribution of TGBp3:H6 in step sucrose gradient as analyzed by silver staining. Right panel, distributions of phospholipids and TGBp3:H6 in the step sucrose gradient. Pink triangle and blue solid circle indicate phospholipids and TGBp3:H6, respectively. (TIF) [file ppat.1003405.s002.tif]

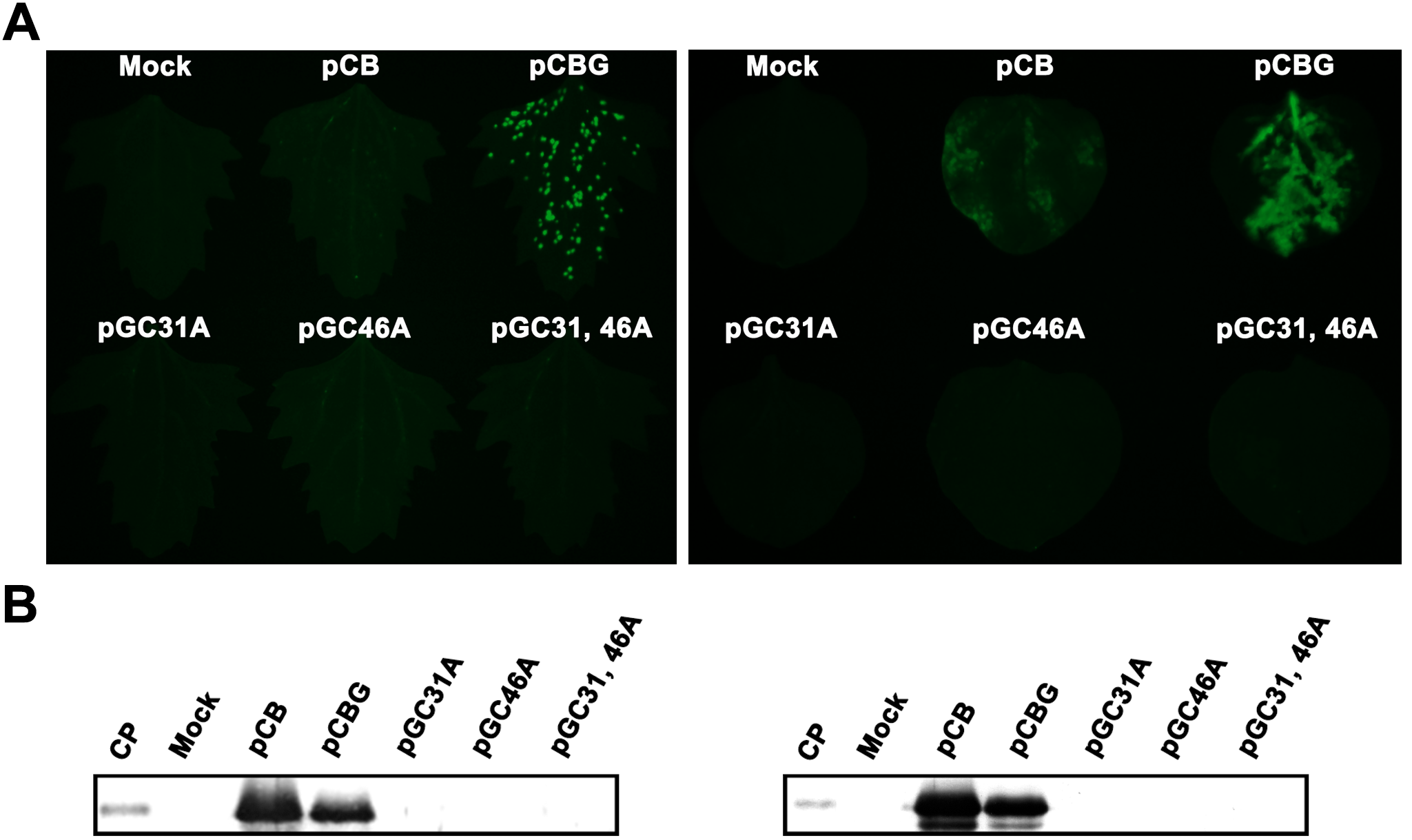

Supplement: Figure S3 — The loss of infectivity of the mutant BaMV with Cys-to-Ala substitution(s) in TGBp3. (A) Effect of Cys-to-Ala substitution in TGBp3 on the infectivity of BaMV. The fluorescence images of leaves were taken 6 dpi for Chenopodium quinoa (left panel) and 17 dpi for Nicotiana benthamiana (right panel). (B) Western blot analysis of CP in plant leaves. The protein samples were extracted from leaves of C. quinoa 10 dpi (left panel) and N. benthamiana 17 dpi (right panel). Protein samples equivalent to 80 µg of inoculated leaves were used for the analysis of CP. The CP sample in the left-most lane of both panels was purified from an Escherichia coli overexpression system. (TIF) [file ppat.1003405.s003.tif]

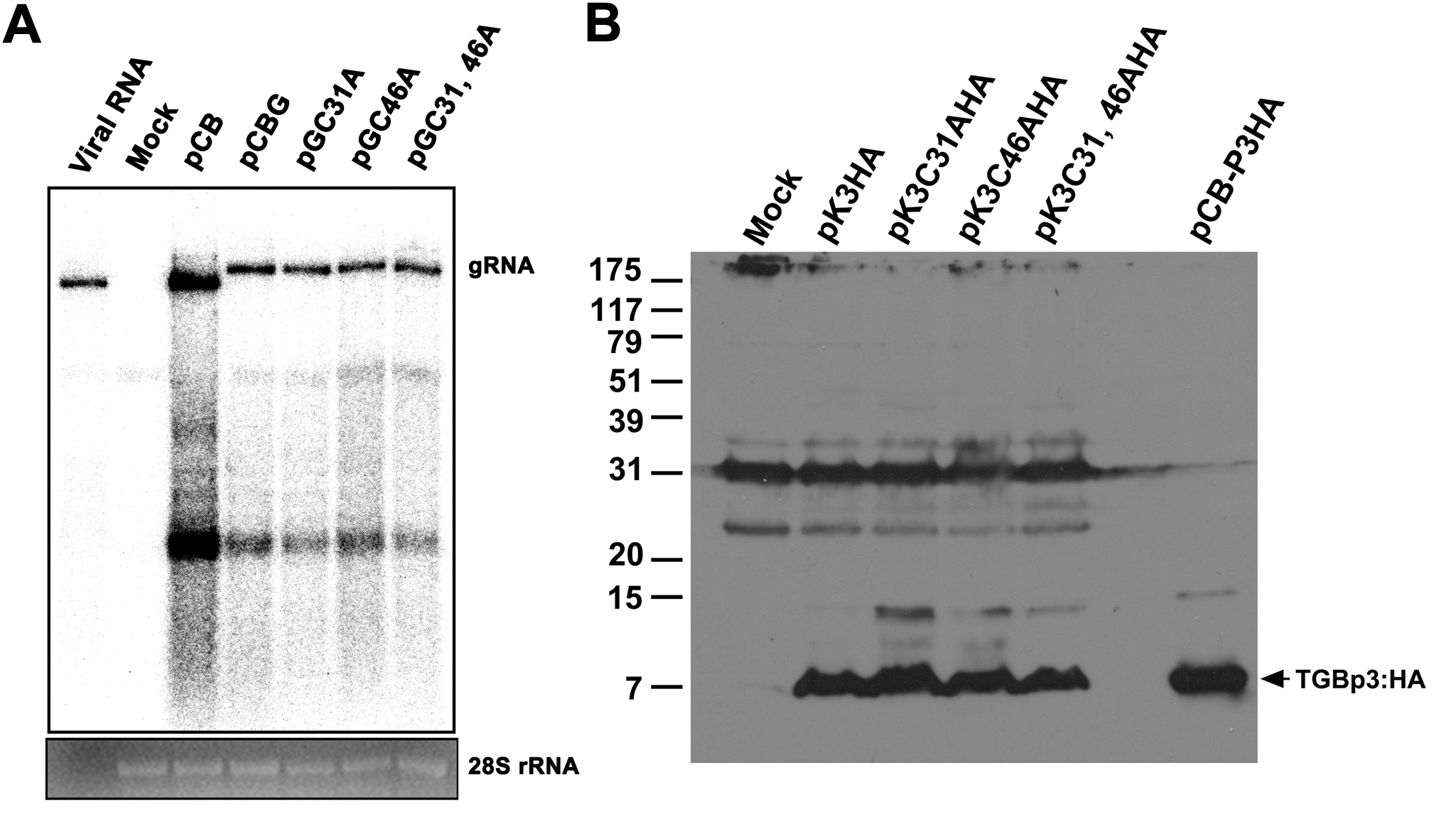

Supplement: Figure S4 — The mutant BaMV with Cys-to-Ala substitution(s) in TGBp3 is active in replication and TGBp3 expression. (A) Northern blot analysis of viral RNA in protoplasts of N. benthamiana. Total nucleic acids extracted from protoplasts infected with each of the plasmid clones of BaMV were separated in a 1.2% agarose gel, blotted onto a nylon membrane, and probed with a 600-base long 32P-labeled RNA complementary to the 3′ end of BaMV genomic RNA. Viral RNA in the left-most lane was purified from C. quinoa inoculated with the infectious WT plasmid clone of BaMV, pCB. The full-length genomic RNA (gRNA) and internal control (28S ribosomal RNA [rRNA]) are shown. (B) Contents of the WT and mutant TGBp3:HA in N. benthamiana as analyzed by western blot. M and D indicate the monomeric and dimeric forms of TGBp3:HA, respectively. (TIF) [file ppat.1003405.s004.tif]
