# Supplementary material for: The Stable Association of Virion with the Triple-gene-block Protein 3-based Complex of Bamboo mosaic virus
Source: PLoS Pathog. 2013 Jun 6;9(6):e1003405. doi: 10.1371/journal.ppat.1003405 (PMC3675025; doi:10.1371/journal.ppat.1003405)
Supplement: Text S1 — Details, including Results as well as Materials and Methods , for Figures S1, S2, S3 and S4. (DOC) [file ppat.1003405.s005.doc]

**Supporting Information (Text S1)**

**Results**

Expression and purification of TGBp3 with a His-tag fusion at its C-terminus

Since the membrane sample prepared from BaMV-infected tissues was not pure enough for further biochemical characterization of TGBp3, we therefore prepared proteoliposomes containing exclusively TGBp3. To start with, we tried to express the C-terminally His-tagged BaMV TGBp3 (TGBp3:H6) in *Escherichia coli* (Figure S1A). As shown in Figure S1B, a protein with a gel mobility as expected for TGBp3:H6 and reacting with anti-His-tag was observed in *E. coli* Rosetta (DE3)/pLysSRARE (p3cH6) but not in *E. coli* BL21(DE3) (p3cH6), suggesting that the high percentage (25%) of rare codons in TGBp3:H6 might have affected its expression in *E. coli*. The putative TGBp3:H6 was further purified with Talon resin, recovered from Tricine SDS-polyacrylamide gel and subjected to N-terminal sequencing. The first 8 amino acid residues obtained were the same as those expected for the recombinant TGBp3:H6 (having an extra Ala (A) residue inserted between the most N-terminal two residues, Met (M) and Leu (L), of TGBp3:HA as shown in Figure 1A), confirming that TGBp3:H6 was really overexpressed. Moreover, we found that the TGBp3:H6 purified from the Talon resin has a tendency to form oligomer even in the presence of SDS and β-mercaptoethenol (Figure S1C), suggesting that the transmembrane helix of TGBp3:H6 is prone to self aggregation.

Reconstitution of TGBp3:H6-containing proteoliposomes *in vitro*

In order to obtain TGBp3:H6 as pure as possible for *in vitro* proteoliposome reconstitution, the TGBp3:H6 purified from Co^2+^-resin (Figure S1C) was further separated on Tricine SDS-polyacrylamide gel and recovered by electroelution. To reconstitute TGBp3:H6-containing proteoliposomes *in vitro*, a micelle-vesicle transition method [1] was adopted. Successful reconstitution of TGBp3:H6-containing proteoliposomes was confirmed by co-fractionation of TGBp3:H6 with phospholipids during 1-10% linear Ficoll gradient centrifugation. As shown in Figure S2A, the reconstituted liposomes containing phospholipids only (left panel) and the TGBp3:H6 solubilized with buffer containing 1% Triton X-100 alone (middle pane) were sedimented mainly in fractions 10-12 and fractions 1-5 of the gradient, respectively. However, the TGBp3:H6-containing proteoliposomes were mostly sedimented in fractions 11-15 of the gradient as evidenced by the presence of both phospholipids and TGBp3:H6 in those fractions (right panel), suggesting that TGBp3:H6 is associated with lipid bilayers after proteoliposome reconstitution. The association of TGBp3:H6 with lipid bilayers can also be seen with flotation assay [2,3]. In this assay, the TGBp3:H6-containing proteoliposomes seemed to condense in the interface between 0% and 25% (w/v) of sucrose (Figure S2B, left panel). This observation was confirmed by silver staining analysis and phosphate assay of the samples collected from the step sucrose gradient, in which most TGBp3:H6 (Figure S2B, middle panel) and phospholipids (Figure S2B, right panel) were present in the top fractions of 25% sucrose.

Replacement of Cys-31 and/or Cys-46 with alanine in TGBp3 results in loss of infectivity of BaMV

To clarify that the two highly conserved cysteine residues, Cys-31 and Cys-46, in the C-terminal tail of TGBp3 play a role in virus movement, the mutant BaMV clones (pGC31A, pGC46A and pGC31, 46A) and their WT counterparts (pCB and pCBG) were then inoculated onto leaves of *Chenopodiun quinoa* and *Nicotiana benthamiana*, respectively. Since the WT and mutant BaMV all contain an expressible green fluorescent protein (GFP) gene in the genome (Figure 9A), green fluorescence spread in inoculated leaves was expected if the tested BaMV has the ability to replicate and move from cell to cell. The spread of green fluorescence in leaves of *C. quinoa* and *N. benthamiana* was examined 6 days post-inoculation (dpi) and 17 dpi, respectively. No fluorescence spread (or disease symptom) (Figure S3A) and no CP (Figure S3B) was detected in *C. quinoa* (left panel) and *N. benthamiana* (right panel) leaves inoculated with any one of the three mutant BaMV. In contrast, green fluorescence spread (or disease symptom) (Figure S3A) and CP (Figure S3B) were detected in both plant leaves inoculated with the WT counterparts. Apparently, the three mutant BaMV have severely lost their infectivity.

The mutant BaMV with Cys-to-Ala substitution(s) in TGBp3 is active in replication and TGBp3 expression

To explore the reason responsible for the loss of infectivity of the mutant BaMV containing Cys-to-Ala substitution in TGBp3, we first examined the replication activities of the mutant BaMV in protoplast using Northern blot analysis (Figure S4A). Similar contents of the full-length genomic RNA of BaMV were detected in protoplasts inoculated with pCBG or each of the three mutant BaMV, indicating that the Cys-to-Ala substitution in TGBp3 does not affect the replication activity of BaMV. Thus, other reasons such as that the Cys-to-Ala substitution might make the expression (sum total of TGBp3 synthesis and degradation) of TGBp3 abnormal or make TGBp3 defective in assisting virus movement were considered.

To clarify whether the Cys-to-Ala substitution in TGBp3 is detrimental to TGBp3 expression, we first compared the contents of WT and mutant TGBp3:HA in leaves infected with the satBaMV-derived plasmid, p3HA, p3C31AHA, p3C46AHA or p3C31, 46AHA, along with pCB, by western blot. However, it was unsuccessful due to low expression of TGBp3:HA. To improve TGBp3:HA expression, the agro-compatible pKn-derived plasmids, pK3HA, pK3C31AHA, pK3C46AHA and pK3C31, 46AHA, harboring the gene of either the WT TGBp3 or the Cys-to-Ala substitution mutant of TGBp3 were constructed and introduced into leaves of *N. benthamiana* by agroinfiltration. Similar levels of TGBp3:HA were observed in *N. benthamiana* leaves after agroinfiltration (Figure S4B). These results clearly indicated that the loss of infectivity of the mutant BaMV is irrelevant to TGBp3 expression.

**Materials and Methods**

Overexpression and purification of BaMV TGBp3:H6

To obtain the TGBp3:H6-overexpressing clone, pET-p3cH6, the BaMV TGBp3 gene was amplified with forward and reverse primers 5'- AGACTACCATGGCTCTAA ACACTGACACACTATGC-3' and 5'-GTGGTGCTCGAGGCTGGAGGTGGTGTGGTAG-3', respectively. The PCR product was cloned into compatible sites of pET21d plasmid after *Nco* I and *Xho* I digestion. The resultant plasmid, pET-p3cH6, was able to overexpress TGBp3:H6 in *E. coli* Rosetta (DE3)/pLysSRARE, and the TGBp3:H6 were insoluble and associated with the membrane fraction of *E. coli* cells. The purify the overexpressed TGBp3:H6, the *E. coli* cell pellet from one liter liquid culture was suspended with buffer L-T (50 mM sodium phosphate, pH 7.0, 0.5 M NaCl, 5% glycerol, 1% Triton X-100, 1 mM PMSF, 100 μg DNase I, 100 μg RNase A, 1 mg/ml Lysozyme) and lysed with a hand homogenizer. The cell homogenate was centrifuged at 30,000 ***g***, 4°C for 30 min. The resultant pellet was resuspended with binding buffer (50 mM sodium phosphate, pH 7.0, 0.3 M NaCl, 5% glycerol, 6 M guanidine hydrochloride, 0.1 mM PMSF), homogenized with a hand homogenizer, and centrifuged at 80,000 ***g***, 4°C for 30 min. The supernatant thus obtained was passed through a 0.45 μm syringe filter and purified by a TALON superflow column. The eluate containing TGBp3:H6 was diluted at least two folds ddH_2_O and precipitated with 4 volumes of –20°C-chilled 100% acetone. The TGBp3:H6 in the pellet were then separated on Tricine SDS-PAGE, stained with a 4°C-cold 0.25 M KCl solution, cut out from the gel, and recovered by electroelution. The gel-purified TGBp3:H6 were precipitated with 4 volumes of –20°C-chilled 100% acetone, washed 4 times with a solution containing 80% acetone and 20% buffer A' (50 mM Hepes, pH 7.4, 1 mM EDTA, 0.15 M NaCl, 1 mM DTT, and 1 mM PMSF) and kept at –20°C in 80% acetone solution until being used.

Preparation of TritonX-100-solubilized TGBp3:H6

To solubilize and refold TGBp3:H6, the acetone pellet of TGBp3:H6 was vacuum-dried, dissolved in buffer H (20 mM Hepes, pH 7.4, 0.1 mM EDTA, 50 mM NaCl, 1 mM DTT, and 1 mM PMSF) supplemented with 6 M guanidine hydrochloride (Gn-HCl) and 1% (w/v) of Triton X-100, dialyzed against buffer H containing 1% (w/v) of Triton X-100 to remove Gn-HCl and allow refolding of TGBp3:H6. The refolded TGBp3:H6 sample was then centrifuged at 4°C, 7,800 ***g*** for 10 min to remove insoluble impurities.

Preparation of lipid-detergent mixed micelles

To prepare lipid-detergent mixed micelles, 30 mg of asolectin (Fluka) dissolved in 3 ml of cyclohexane was placed into a round bottom flask and dried with a rotary evaporator on a 25°C water bath. This operation produced a fine asolectin film on the wall of the flask. The residual cyclohexane in the film was then removed by vacuum evaporation. The asolectin film was dissolved with 3 ml of buffer H containing 1% (w/v) of Triton X-100. The sample containing lipid-detergent mixed micelles was then passed through a 0.2 μm Acrodisc syringe filter before being used for *in vitro* proteoliposome reconstitution. The concentration of phospholipids in the filtrate was about 10 mg/ml.

*In vitro* reconstitution of proteoliposomes containing TGBp3:H6

To reconstitute the TGBp3:H6-containing proteoliposomes, 25 μg of the Triton X-100-solubilized TGBp3:H6 was mixed with asolectin-Triton X-100 mixed micelles in a volume of 1 ml (the weight ratio of TGBp3:H6 to asolectin was 1:200). The mixture was incubated at 4°C for 30 min on a rotation mixer and then moved to a microcentrifuge tube containing buffer H-equilibrated Bio-beads SM-2 (Bio-Rad) at room temperature for 1 h to facilitate the removal of Triton X-100. The Triton X-100 in the mixture was further adsorbed with two changes of fresh Bio-beads by incubation at 4°C on the same rotation mixer overnight. After Bio-beads adsorption, the reconstituted TGBp3:H6-containing proteoliposomes were layered on top of a linear Ficoll (Sigma, type 400) gradient (0-10%, w/v) in buffer H and centrifuged at 110,000 ***g***, 4°C for 20 h in a Hitachi RPS40T swing bucket rotor. After centrifugation, the gradient sample was fractionated into 500 μl fractions from top to bottom. The contents of protein and phospholipid in each fraction were analyzed by phosphate assay and Tricine SDS-PAGE.

Phosphate assay

Phosphate assay [4] was used to measure the contents of phospholipids in the fractionated samples. Briefly, 50 μl of ashing buffer, 10% Mg(NO_3_)_2_ in ethanol, was added into 100 μl of proteoliposome sample from each fraction. After mixing, the sample was baked with an oven at 550˚C for 4 h to white ash, which was then solubilized with 0.3 ml of 1 N HCl and heated in boiling water for 15 min. After cooling to room temperature, the sample was mixed with 100 μl of 10% ascorbic acid and 600 μl of 0.42% ammonium molybdate in 1 N sulfuric acid. The resultant sample was incubated at 45˚C for 20 min before measuring the optical density at 820 nm and the content of phosphate was obtained by calibrating with a standard curve obtained by replacing the gradient sample with 3 to 30 μl of 1 mM NaH_2_PO_4_ solution.

Purification of the reconstituted TGBp3:H6-containing proteoliposomes by flotation

For fast and efficient purification of TGBp3:H6-containing proteoliposomes, flotation assay [5] was performed. Briefly, 400 μl of the reconstituted TGBp3:H6-containing proteoliposomes was mixed with 350 μl of 75% (w/v) sucrose to a final concentration of 30% (w/v) sucrose in an ultracentrifuge tube. Then, the sample was overlaid with 450 μl of buffer H containing 25% (w/v) sucrose and 150 μl of buffer H. The sample was further centrifuged at 240,000 ***g*** in a Hitachi swing rotor (S55S) for 1.5 h, and fractionated into 150 μl fractions from top to bottom. The contents of protein and phospholipid in each fraction were analyzed by phosphate assay and Tricine SDS-PAGE.

Construction of plasmid, pK3HA, pK3C31AHA, pK3C46AHA or pK3C31, 46AHA, expressing WT or mutant TGBp3:HA in *N. benthamiana*.

To construct agro-compatible plasmid expressing the WT or mutant TGBp3 with an HA fusion at the C-terminal end, the DNA coding for the WT or mutant TGBp3:HA was recovered from the plasmid, p3HA, p3C31AHA, p3C46AHA or p3C31, 46AHA, after digestion with *Hind*III and *Bgl*II. The recovered DNA was then cloned into the compatible sites on pKn. The resultant plasmids were named pK3HA, pK3C31AHA, pK3C46AHA or pK3C31, 46AHA.

Identification of proteins associated with the TGBp3-containing membrane complex by LC-MS-MS

The co-immunoprecipitated protein sample (IpP) obtained by treating the Protein A-Sepharose pretreated S100 with anti-HA (see Materials and Methods in the main text) was run on a Tricine SDS-polyacrylamide gel and subjected to silver staining. The P25 and P28 protein bands as observed in lanes 6, 8 and 10 of Figure 4B were excised from the gel and subjected to in gel digestion with trypsin. The tryptic fragments thus obtained were separated with a LC system (nanoACQUITY UPLC and autosampler system, Waters) before sequence analysis by an ESI-QUAD-TOF (QSTAR Elite, Applied Biosystems Sciex).

**References**

1. Hsu HT, Chou YL, Tseng YH, Lin YH, Lin TM, et al. (2008) Topological properties of the triple gene block protein 2 of Bamboo mosaic virus. Virology 379: 1-9.
2. Futai E, Hamamoto S, Orci L, Schekman R. (2004) GTP/GDP exchange by Sec12p enables COPII vesicle bud formation on synthetic liposomes. EMBO J 23: 4146-4155.
3. Weber T, Zemelman BV, McNew JA, Westermann B, Gmachl M, et al. (1998) SNAREpins: minimal machinery for membrane fusion. Cell 92: 759-772.
4. Fiske CM, Subbarow, Y. (1925) The colorimetric determination of phosphorus. J Biol Chem 66: 375-400.
5. Bigay J, Casella JF, Drin G, Mesmin B, Antonny B. (2005) ArfGAP1 responds to membrane curvature through the folding of a lipid packing sensor motif. EMBO J 24: 2244-2253.
